# Psychosocial interventions for post-traumatic stress disorder in refugees and asylum seekers resettled in high-income countries: Systematic review and meta-analysis

**DOI:** 10.1371/journal.pone.0171030

**Published:** 2017-02-02

**Authors:** Michela Nosè, Francesca Ballette, Irene Bighelli, Giulia Turrini, Marianna Purgato, Wietse Tol, Stefan Priebe, Corrado Barbui

**Affiliations:** 1 WHO Collaborating Centre for Research and Training in Mental Health and Service Evaluation, Department of Neuroscience, Biomedicine and Movement Sciences, Section of Psychiatry, University of Verona, Verona, Italy; 2 Department of Mental Health, Johns Hopkins Bloomberg School of Public Health, Baltimore, Maryland, United States of America; 3 Unit for Social and Community Psychiatry, WHO Collaborating Centre for Mental Health Services Development, Queen Mary University of London, London, United Kingdom; Central Institute of Mental Health, GERMANY

## Abstract

Treatment of post-traumatic stress disorder (PTSD) in refugees and asylum seekers resettled in high-income countries presents specific challenges. This systematic review examined the effectiveness of psychosocial interventions for this group. We searched the Cochrane Central Register of randomised trials, CINAHL, EMBASE, PILOTS, PsycINFO, PubMed and Web of Science up to July 2016. Studies included randomised and controlled clinical trials comparing psychosocial interventions with waiting list or treatment as usual in adult refugees and asylum seekers with PTSD resettled in high-income countries. PTSD symptoms post-intervention was the primary outcome. We computed standardized mean differences (SMD) with 95% confidence intervals (CI). This study is registered with PROSPERO: CRD42015027843. Twelve studies were included in the meta-analysis. Psychosocial interventions were effective in decreasing PTSD symptoms relative to control groups (SMD -1·03, 95% CI -1·55 to -0·51; number needed to treat 4·4; I^2^ 86%; 95% CI 77 to 91). Narrative exposure therapy, a manualized short-term variant of cognitive behavioural therapy with a trauma focus, was the best-supported intervention (5 RCTs, 187 participants, SMD -0·78, 95% CI -1·18 to -0·38, *I*^*2*^ 37%; 95% CI 0 to 77). Methodological quality of the included studies was limited. Overall, psychosocial interventions for asylum seekers and refugees with PTSD resettled in high-income countries were found to provide significant benefits in reducing PTSD symptoms. Yet, the number of studies is small and their methodological quality limited, so that more rigorous trials should be conducted in the future.

## Introduction

Worldwide, around 65 million people are forcibly displaced because of conflict and persecution, including 21.3 million refugees and over 3 million individuals awaiting resolution of their asylum application. Asylum applications in industrialised countries have significantly increased with 80% of these being lodged in European countries [1]. In 2015, the United Nations High Commissioner for Refugees (UNHCR) reported over one million refugees reaching Europe by sea, and a further 34,000 crossing from Turkey into Bulgaria and Greece by land [2].

Traumatic events, such as torture and war exposure, are disproportionately experienced by refugees and asylum seekers before and during displacement [3,4]. In addition, post-displacement traumatic events important for mental health include resettlement stress [5], and perceived stigma and discrimination [6,7]. In comparison with the general population, refugees and asylum seekers have been shown to experience higher prevalence rates of a range of disorders, including common mental disorders (e.g. depression, anxiety, somatoform disorders), severe mental disorders (e.g. psychosis), substance use disorders, and disorders specifically tied to stress [8–10]. Despite the range of mental disorders of relevance to conflict-affected populations, the best studied mental health outcome in refugees remains post-traumatic stress disorder (PTSD). PTSD is 10 times more likely in refugees and asylum seekers compared to host populations [8–10].

Treatment of PTSD among refugees and asylum seekers resettled in high-income countries presents complex and specific challenges [11]. It can be difficult to distinguish expected temporary distress from PTSD in people who are continuously exposed to various traumatic events. In addition, refugees from various socio-cultural settings may have diverging perspectives on causes and priorities with regard to experienced distress, as compared to the PTSD diagnosis conceptualized in psychiatric classification systems. Trauma experienced by refugees is different in character, severity and duration than that seen in other populations [12–15], leading to the expression of psychopathology with a long-term fluctuating course and a high comorbidity with other disorders, particularly depression [16,17]. In addition, in high-income countries complications with cultural and language barriers, difficulty in developing trust in staff-patient relationships, and increased risk of social marginalisation represent major treatment and social issues [18].

Despite a growing body of literature, including several narrative and systematic reviews that suggested a promising role for some psychosocial interventions [19–25], so far the efficacy of psychosocial interventions on PTSD outcomes in asylum seekers and refugees resettled in high-income countries has never been quantified. Against this background, this study aimed to establish whether the current evidence supports the provision of psychosocial interventions for PTSD in this group. We conducted a systematic review of randomised controlled trials (RCTs) and controlled clinical trials (CCTs) in adult refugees and asylum seekers with PTSD resettled in high income countries.

## Materials and methods

The protocol for this review was registered in the International Prospective Register of Systematic Reviews (PROSPERO), registration number: CRD42015027843.

### Identification and selection of studies

The following bibliographical databases were searched up to July 2016: Cochrane Central Register of randomised trials (CENTRAL), CINAHL, EMBASE, PILOTS, PsycINFO, PubMed and Web of Science. Terms indicative of PTSD were combined with terms indicative of psychosocial interventions (both MeSH terms and text words). Studies in any language were considered for inclusion. Grey literature was searched using the databases listed in the Cochrane Handbook for Systematic Reviews of Interventions [26]. We also checked the references of narrative reviews of psychosocial treatment of PTSD in displaced populations. Details of the searches and of the screening process are given in S1 and S2 Tables. The selection process was recorded in agreement with the Preferred Reporting Items for Systematic Reviews and Meta-Analyses (PRISMA) [27].

The inclusion criteria were: (a) RCT or CCT; (b) the effects of a psychosocial intervention were investigated; (c) psychosocial interventions were compared with no psychosocial intervention, a waiting list, treatment as usual, usual care, repeated assessment, or a minimal attention control group akin to psychological placebo; (d) participants were adult (18 years or above) refugees or asylum seekers with PTSD resettled in high-income countries. The terms ‘psychosocial intervention’, ‘asylum seeker’, ‘refugee’ and ‘high-income country’ are defined in S3 Table.

### Outcome measures

The primary outcome of this review was the mean score post-intervention on the Clinician-Administered PTSD Scale (CAPS) or Harvard Trauma Questionnaire (HTQ) or on any other PTSD rating scale with evidence of adequate validity and reliability. Secondary outcomes were the following: depressive symptoms, as measured by the Hamilton Depression Rating Scale (HDRS), Montgomery-Asberg Depression Rating Scale (MADRS), Clinical Global Impression Rating scale (CGI), or any other depression rating scale with evidence of adequate validity and reliability; PTSD symptoms at follow-up; number of individuals fulfilling PTSD diagnostic criteria at follow-up; number of patients who dropped out during the trial by any cause; and global functioning, as measured by any validated rating scale.

### Data extraction and quality assessment

Data extraction was performed in agreement with the Cochrane Handbook for Systematic Reviews of Interventions, Chapter 7.19 [26]. Two review authors (MN and FB), independently extracted the data on participant characteristics, intervention details and outcome measures. Disagreements were resolved by discussion and consensus with a third member of the team (CB). For continuous outcomes, the mean scores at end-point or the mean change from baseline to end-point, the standard deviation or standard error of these values, and the number of patients included in these analyses, were extracted. For dichotomous outcomes, the number of individuals fulfilling PTSD diagnostic criteria at the end of the study, the number of patients leaving the study early and the number of patients undergoing the randomisation procedure were recorded. When outcome data were not reported, trial authors were asked to supply the data.

The quality of included studies was assessed using the ‘Risk of bias’ assessment tool developed by the Cochrane Collaboration [26]. This tool assesses possible sources of bias in clinical trials, including the adequate generation of allocation sequence; the concealment of allocation to conditions; the prevention of knowledge of the allocated intervention (blinded outcome assessment); and dealing with incomplete outcome data (this was assessed as positive when intention-to-treat analyses were conducted, meaning that all randomized patients were included in the analyses). Emerging recommendations from the Cochrane Collaboration Non-Randomised Studies Methods Group were followed to rate the quality of clinical trials that did not employ a random allocation procedure. Quality assessment was conducted by two independent review authors (MN and IB), and disagreements were solved through discussion.

### Data synthesis

Data entry and analysis was performed twice. First, data were entered and analysed with Review Manager (RevMan 5.3), software recommended by the Cochrane Collaboration, and then independently re-entered into a spreadsheet and analysed within the metan module in STATA 14.1. Statistical outputs were cross-checked for consistency. In accordance with recent efforts towards a data sharing culture [28–29], the spreadsheet with the full dataset is made available as part of this publication.

For continuous outcomes, we pooled the standardised mean differences (SMDs) as different measurement scales were used. A loose intention-to-treat (ITT) analysis was applied, whereby all participants with at least one post-baseline measurement were represented by their last observations carried forward (LOCF) [26]. When only P or standard error values were reported, standard deviations were calculated according to Altman and Bland [30]. If standard deviations could not be calculated, they were imputed using validated methodology [31]. Because some studies had relatively small sample sizes we corrected the effect size for small sample bias, using Hedge’s g [26]. For dichotomous outcomes a Mantel–Haenszel risk ratio was calculated. Continuous and dichotomous outcomes were analysed using a random-effects model, with 95% confidence intervals (CI), as this takes into account any difference between studies even if there is no statistically significant heterogeneity. To provide a measure of clinical significance, the number-needed-to-treat (NNT) was calculated [32].

Studies that compared two or more formats of similar psychosocial interventions were included in meta-analysis by combining group arms into a single group, as recommended in section 16.5 of the Cochrane Handbook [26].

We calculated the *I*^*2*^-statistic, which quantifies the effect of statistical heterogeneity, providing a measure of the degree of inconsistency in the studies' results in percentages [26]. We calculated 95% CIs around *I*^*2*^, using the non-central chi-squared-based approach within the heterogi module in STATA 14.1.

For PTSD and depressive symptomatology, the following subgroup analyses were performed: type of psychosocial intervention (narrative exposure therapy [NET] vs. cognitive behavioral therapy [CBT] vs. eye movement desensitization and reprocessing [EMDR] vs. trauma-focus psychotherapy [TFP] vs. culture-sensitive oriented peer [CROP] vs. family group intervention [FGI]), study design (RCTs vs. CCT), outcome measure (CAPS or HDRS vs. any other instrument), number of sessions (up to 10; between 11 and 20; more than 20), length of follow-up (up to 4 months; more than 4 months), study country (Germany vs. USA vs. other EU countries), and country of origin (one country vs. two or more countries). As number of sessions and length of follow-up were arbitrarily categorised, we further investigated a potential association between effect size and these continuous variables by means of unrestricted maximum likelihood random effects meta-regression analysis, as implemented in Comprehensive Meta-analysis (CMA) [33].

To investigate the impact of each study on the pooled effect (sensitivity analysis), we consecutively removed each study as a possible outlier to test its impact on the combined effect, as implemented in CMA [34].

Publication bias was tested by visually inspecting the funnel plot on primary outcome measures and by Duval and Tweedie’s trim and fill procedure, which yields an estimate of the effect size after the publication bias has been taken into account (as implemented in CMA). Egger’s test of the intercept was conducted to quantify the bias captured by the asymmetry of the funnel plot and test whether it was significant.

In order to produce a tabular synoptic overview of the main review findings and quality, easily understandable for patients, policy makers, research planners, guideline developers and other stakeholders, data were summarised according to the methodology described by the GRADE working group [35]. We followed the WHO criteria for summarising and aggregating the evidence [36–37].

## Results

### Characteristics of included studies

**Fig 1:**
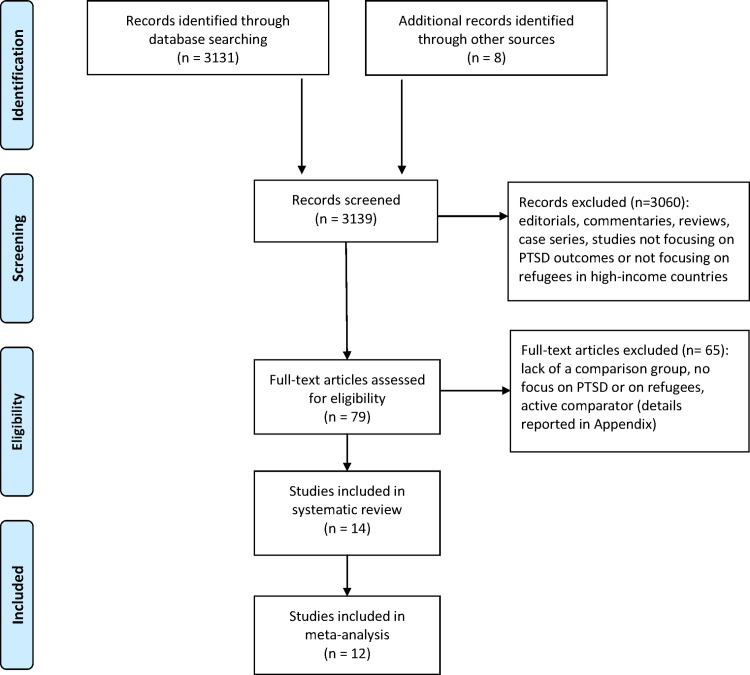
PRISMA flow-chart diagram.

The electronic search yielded a total number of 3139 abstracts (after removal of duplicates). After title and abstract screening, 79 full text papers were considered for inclusion, of which 14 studies met the inclusion criteria and were included in the systematic review [38–51] (Fig 1 and S1 Table). References to excluded studies are reported in S4 Table. Of the included studies, two provided no data that could be used in the meta-analysis.

Of the 14 included studies, 12 were RCTs [38,39,41–43,45–51], and two were CCTs [40,44]. Nine studies had a waiting list control condition, while five compared a psychosocial intervention with treatment as usual (Table 1). The mean study sample size was 63 participants (range 10 to 197), and the mean length of follow-up was five months (range 2 to 12) (Table 1). Nine studies were conducted in Europe and five in the United States of America. Study participants were from a single country in seven studies, while a mix of participants from more than one country was included in the remaining studies. No studies were carried out within newly arrived asylum seekers; time since resettlement ranged from 2 to 16 years.

**Table 1:** Selected characteristics of included studies.

Study	Country	Country of origin	Intervention (No of sessions)	Control	N	Design	PTSD measure	Follow-up (months)
Adenauer 2011	Germany	Africa, Middle East, Balkans	NET (12)	Waiting list	33	RCT	CAPS	3
Buhmann 2016	Denmark	Iran, Iraq, Afghanistan, Balkans	CBT (16)	Waiting list	142	RCT	HTQ	6
Drozdek 2010	Netherlands	Iran, Iraq, Afghanistan	TFP (85)	Waiting list	82	CCT	HTQ	12
Hijazi 2014	USA	Iraq	NET (3)	Waiting list	63	RCT	HTQ	2
Hinton 2004	USA	Vietnam	CBT (11)	Waiting list	12	RCT	HTQ	3
Hinton 2005	USA	Cambodia	CBT (12)	Waiting list	40	RCT	HTQ	3
Kruse 2009	Germany	Bosnian	TFP (25)	TAU	70	CCT	HTQ	12
Liedl 2011	Germany	Balkans, Turkey	CBT (10)	Waiting list	36	RCT	PDS	3
Morath 2014	Germany	Africa, Middle East	NET (12)	Waiting list	34	RCT	CAPS	4
Neuner 2010	Germany	Turkey, Balkans, Africa	NET (9)	TAU	32	RCT	PDS	8
Otto 2003	USA	Cambodia	CBT (10)	TAU	10	RCT	CAPS	
Renner 2011	Austria	Chechnya	CROP (16)	Waiting list	56	RCT	HTQ	4
Stenmark 2013	Norway	Iraq, Afghanistan, Africa, Middle East	NET (10)	TAU	81	RCT	CAPS	6
Weine 2008	USA	Bosnia	FGI (9)	TAU	197	RCT		6

Abbreviations: NET: Narrative Exposure Therapy; CBT. Cognitive Behavior Therapy; TFP: Trauma Focused Psychotherapy; CROP: Culture-Sensitive Oriented Peer; FGI: Family-Group Intervention; TAU: Treatment as usual; RCT: Randomised Controlled Trial, CCT: Controlled Clinical Trial; PTSD: Posttraumatic stress disorder; CAPS: Clinician-Administered PTSD Scale; HTQ: Harvard Trauma questionnaire, PDS: Post Traumatic Stress Diagnostic scale.

Interventions were all psychological: NET, a manualized short-term variant of CBT with a trauma focus (five RCTs), other types of CBT (five RCTs), TFP (two CCTs), CROP (one RCT), and FGI (one RCT) (Table 1). Four studies assessed group interventions [40,48,49,51], of which two were included in the analysis [40,49]. Study participants were exposed to an average number of 17 face-to-face sessions (range 3 to 25) of psychosocial interventions. The main characteristics of these interventions are described in S5 Table.

**Fig 2:**
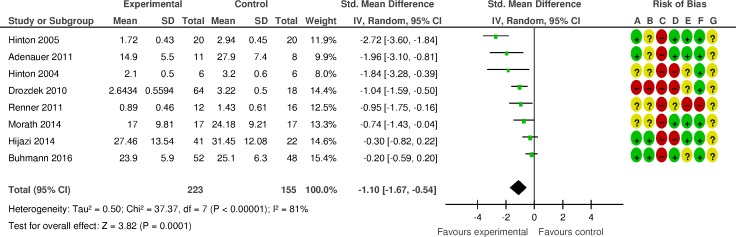
Efficacy of psychosocial interventions for PTSD in refugees and asylum seekers displaced in high-income countries: PTSD symptoms. (A) Random sequence generation; (B) Allocation concealment; (C) Blinding of participants and personnel; (D) Blinding of outcome assessment; (E) Incomplete outcome data; (F) Selective reporting; (G) Other bias.

The quality of the studies varied (Fig 2 and S6 Table). No study met all seven quality criteria. Four of five studies on NET included authors who developed the NET manual. Using the GRADE framework, the quality of evidence ranged between low and very low (S7 Table).

### Effects of psychosocial interventions compared to control: Primary outcome

The meta-analysis of the primary outcome (12 studies, 543 patients) showed that psychosocial interventions were effective in decreasing PTSD symptoms relative to inactive controls (SMD -1·03, 95% CI -1·55 to -0·51) (Fig 2). The magnitude of effect corresponds to a NNT of 4·4. Heterogeneity was high (*I2* = 86%; 95% CI 77 to 91). Visual inspection of the funnel plot and Egger’s test (*p* = ·04) suggested the possibility of publication bias (S1 Fig). Removing each of the studies as a possible outlier, including one RCT that was retracted for administrative but not scientific abnormalities (S6 Table), resulted in no change in the overall estimate (S2 Fig).

### Effects of psychosocial interventions compared to control: Secondary outcomes

**Fig 3:**
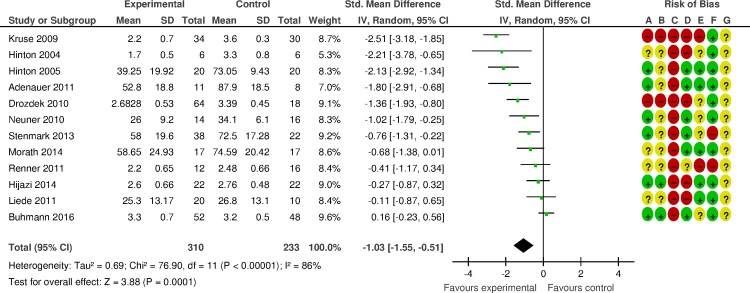
Efficacy of psychosocial interventions for PTSD in refugees and asylum seekers displaced in high-income countries: depressive symptoms. (A) Random sequence generation; (B) Allocation concealment; (C) Blinding of participants and personnel; (D) Blinding of outcome assessment; (E) Incomplete outcome data; (F) Selective reporting; (G) Other bias.

The meta-analysis of depressive outcomes (eight studies, 378 patients) showed that psychosocial interventions were effective in decreasing depression relative to inactive controls (SMD -1·10, 95% CI -1·67 to -0·54) (Fig 3). The magnitude of effect corresponds to a NNT of 3·1. Heterogeneity was high (*I2* = 81%; 95% CI 64 to 90). Very few studies contributed to the analyses of the other secondary outcomes. In terms of PTSD symptoms at follow-up, psychosocial interventions were not more effective than control groups, although a wide CI around the overall point estimate leaves the possibility of clinically relevant differences (SMD 0·01, 95% CI -1·26 to 1·28). Psychosocial interventions showed a positive effect in terms of participants still fulfilling PTSD criteria at follow-up (three studies, 158 participants, RR 0·39, 95% CI 0·19 to 0·83, *I2* = 84%; 95% CI 52 to 95, NNT = 2), while in terms of dropout rate no differences were observed (S3 Fig). For the secondary outcome global functioning we found no data suitable for meta-analysis.

### Subgroup analyses

In terms of type of psychosocial intervention, subgroup analyses of PTSD outcomes showed that NET was effective in decreasing PTSD symptoms relative to inactive controls (five RCTs, 187 participants, SMD -0·78, 95% CI -1·18 to -0·38, *I2* = 37%; 95% CI 0 to 77). The magnitude of the effect corresponds to a NNT of 6·7 participants (Table 2). Other types of CBT taken together failed to show a significant effect, although the confidence interval around the point estimate did not exclude the possibility of a clinically relevant benefit (Table 2). The magnitude of effect of TFsuppP was substantial, but supported by two CCTs only. CROP was studied in one RCT only and no studies on EMDR were found (Table 2) (S4 Fig).

**Table 2:** Standardized effect sizes of psychosocial interventions for PTSD in refugees and asylum seekers displaced in high-income countries: subgroup analyses of PTSD outcomes.

Meta-analysis	Studies (N)	Patients (N)	SMD	95% CI	*I*^*2*^ ^a)^	95% CI ^b)^	*P* ^c)^	NNT
Overall PTSD outcomes								
All studies	12	543	-1·03	-1·55 to -0·51	86	77 to 91	·00	4·4
Subgroup analyses								
Intervention							·18	
NET	5	187	-0·78	-1·18 to -0·38	37	0 to 77		6·7
CBT	4	182	-0·97	-2·20 to 0·26	91	79 to 96		
EMDR	0							
TFP	2	146	-1·92	-3·05 to -0·80	85	NA		2·1
CROP	1	28	-0·41	-1·17 to 0·34	NA	NA		
Study design							·07	
RCT	10	397	-0·81	-1·28 to -0·33	78	60 to 88		6·3
CCT	2	146	-1·92	-3·05 to -0·80	85	NA		2·1
Study quality							·98	
Low RoB	5	223	-1·04	-1·95 to -0·13	88	75 to 94		4·3
High RoB	7	320	-1·03	-1·69 to -0·37	84	69 to 92		4·4
Rating scale							·47	
CAPS	4	153	-1·28	-1·99 to -0·56	72	21 to 90		3·2
Others	8	390	-0·90	-1·60 to -0·21	89	80 to 94		5·0
No of sessions							·05	
Up to 10	4	164	-0·54	-0·93 to -0·16	28	0 to 73		11·3
11–20	6	233	-1·08	-1·93 to -0·22	87	73 to 93		4·1
More than 20	2	146	-1·92	-3·05 to -0·80	85	NA		2·1
Length of FU							·55	
Up to 4 months	6	188	-0·86	-1·52 to -0·20	76	46 to 89		5·8
More than 4 months	6	355	-1·18	-2·02 to -0·35	91	83 to 95		3·6
Country							·41	
Germany	5	177	-1·22	-2·11 to -0·32	85	66 to 93		3·4
USA	3	96	-1·46	-2·91 to -0·01	87	64 to 96		2·6
Other EU countries	4	270	-0·58	-1·30 to 0·13	85	64 to 94		7·5
Ethnicity							·23	
One country	5	188	-1·46	-2·50 to -0·42	89	76 to 95		2·6
Two or more	7	355	-0·74	-1·27 to -0·21	79	57 to 90		6·3

Abbreviations: PTSD: Posttraumatic stress disorder; NET: Narrative exposure therapy; CBT: Cognitive behavioural therapy; EMDR: Eye movement desensitization and reprocessing; TFP: Trauma focused psychotherapy; CROP: Culture-Sensitive Oriented Peer; RCT: Randomized clinical trial; CCT: Controlled clinical trial; RoB: Risk of bias; CAPS: Clinician-administered PTSD scale; FU: Follow-up; SMD: Standardized mean difference; CI: Confidence interval; NNT: number-needed-to-be-treated.

^a)^ Calculated when at least two studies contributed to the analysis.

^b)^ Calculated when at least three studies (two degrees of freedom) contributed to the analysis.

^c)^ The p-value in this column indicates whether the effect sizes of subgroups differ significantly from each other in the subgroup analyses.

The magnitude of effect was higher in CCTs compared to RCTs, and in studies with more sessions and lasting more than four months (Table 2). Using meta-regression analyses to further investigate the association between number of sessions and effect size (p = 0·44), and between length of follow-up and effect size (p = 0·08), a similar trend was observed (S5 and S6 Figs).

Other subgroup analyses of PTSD outcomes showed no difference between the SMDs in terms of study quality, use of CAPS versus other outcome measures, country where the study was carried out, or ethnicity (Table 2).

Subgroup analyses of depression outcomes (S7 Fig) showed that NET was effective in decreasing depressive symptoms relative to control groups (three RCTs, 116 participants, SMD -0·86, 95% CI -1·65 to -0·06, *I2* = 70%; 95% CI 0 to 91). The magnitude of effect corresponds to a NNT of 4·3 participants (Table 3). Other types of CBT taken together failed to show a significant effect, although the confidence interval around the point estimate did not exclude the possibility of a clinically relevant benefit (Table 3). The effect of TFP and CROP on depressive symptoms was significant, but supported by one small study only. No studies on EMDR were found (Table 3).

**Table 3:** Standardized effect sizes of psychosocial interventions for PTSD in refugees and asylum seekers displaced in high-income countries: subgroup analyses of depressive outcomes.

Meta-analysis	Studies (N)	Patients (N)	SMD	95% CI	*I*^*2*^ ^a)^	95% CI ^b)^	*P* ^c)^	NNT
Overall depressive outcomes								
All studies	8	378	-1·10	-1·67 to -0·54	81	64 to 90	·00	3·1
Subgroup analyses								
Intervention							·92	
NET	3	116	-0·86	-1·65 to -0·06	70	0 to 91		4·3
CBT	3	152	-1·54	-3·38 to 0·29	93	83 to 97		
EMDR	0							
TFP	1	82	-1·04	-1·59 to -0·50	NA	NA		3·3
CROP	1	28	-0·95	-1·75 to -0·16	NA	NA		3·7
Study design							·84	
RCT	7	296	-1·13	-1·81 to -0·46	83	67 to 92		3·0
CCT	1	82	-1·04	-1·59 to -0·50	NA	NA		3·3
Study quality							·45	
Low RoB	4	193	-1·34	-2·49 to -0·19	90	78 to 96		2·4
High RoB	4	185	-0·86	-1·38 to -0·33	53	0 to 84		4·3
Rating scale							·42	
HDRS	3	153	-0·82	-1·67 to 0·03	77	27 to 93		
Others	5	225	-1·29	-2·09 to -0·49	82	60 to 92		2·7
No of sessions							·06	
Up to 10	1	63	-0·30	-0·82 to 0·22	NA	NA		
11–20	6	233	-1·32	-2·15 to -0·49	85	69 to 93		2·4
More than 20	1	82	-1·04	-1·59 to -0·50	NA	NA		3·3
Length of FU							·63	
Up to 4 months	5	177	-1·24	-2·09 to -0·38	83	61 to 92		2·6
More than 4 months	3	201	-0·94	-1·81 to -0·06	83	49 to 94		3·8
Country							·49	
Germany	2	53	-1·26	-2·44 to -0·08	68	NA		2·6
USA	3	115	-1·58	-3·31 to 0·14	91	77 to 97		1·9
Other EU countries	3	210	-0·68	-1·30 to -0·07	72	6 to 92		4·9
Ethnicity							·42	
One country	4	143	-1·40	-2·55 to -0·25	87	68 to 95		2·2
Two or more	4	235	-0·85	-1·48 to -0·22	75	31 to 91		4·9

Abbreviations: PTSD: Posttraumatic stress disorder; NET: Narrative exposure therapy; CBT: Cognitive behavioural therapy; EMDR: Eye movement desensitization and reprocessing; TFP: Trauma focused psychotherapy; CROP: Culture-Sensitive Oriented Peer; RCT: Randomized clinical trial; CCT: Controlled clinical trial; RoB: Risk of bias; HDRS: Hamilton depression rating scale; FU: Follow-up; SMD: Standardized mean difference; CI: Confidence interval; NNT: number-needed-to-be-treated.

^a)^ Calculated when at least two studies contributed to the analysis.

^b)^ Calculated when at least three studies (two degrees of freedom) contributed to the analysis.

^c)^ The p-value in this column indicates whether the effect sizes of subgroups differ significantly from each other in the subgroup analyses.

Other subgroup analyses of depression outcomes showed no difference between the SMDs in terms of study design, quality, use of HDRS versus other outcome measures, number of sessions, length of follow-up, country where the study was carried out, and ethnicity (Table 3).

## Discussion

Considering that since 2008 a total of nearly 1.1 million asylum seekers have been granted a refugee status in Europe, this review may be relevant for the care of nearly 100,000 people with PTSD recently resettled in Europe alone [2]. With respect to PTSD symptoms, the findings suggest that between four and five refugees and asylum seekers with PTSD need to be treated in order for one to benefit. A slightly higher magnitude of effect was observed on depressive outcomes. However, the limited study quality casts uncertainty about these effects. Seven of the 12 studies included in the primary outcome analysis were at high risk of bias in two or more items of the Cochrane risk of bias tool, with no studies being free from bias in all quality items. In particular, in four studies blinding of outcome assessment was not mentioned.

Findings from this review add to the existing literature on the effectiveness of psychosocial interventions in individuals with PTSD who were selected for being forcibly displaced because of conflict and persecution. Crumlish and colleagues, who conducted a qualitative review of randomised controlled trials of treatments of PTSD among refugees and asylum seekers, irrespective of the country in which they resettled, suggested that NET, a manualized short-term variant of trauma-focused cognitive behavioural therapy, was the best-supported modality [19]. A similar conclusion was reached by Gwozdziewycz and Mehl-Madrona, who considered seven studies related to narrative exposure methods for treating trauma in refugees, irrespective of the setting of care [20]. For torture survivors, a population with some commonalities with refugee survivors, a Cochrane review showed that NET and other forms of CBT were found to provide moderate benefits in reducing distress and PTSD symptoms over the medium term, although evidence was of very low quality [21]. Our findings provide further empirical evidence that psychosocial interventions that are effective for PTSD in the general population may not completely overlap with those that are appropriate for PTSD in asylum seekers and refugees in high-income settings. In the general population of adults with PTSD existing guidelines suggest CBT with a trauma focus, EMDR, or stress management [22,23]. By contrast, this systematic review found that some evidence existed in support of NET, that TFP was supported by two heterogeneous studies, and that for other types of CBT taken together the evidence was inconclusive. No studies were found for EMDR. It should be considered, however, that NET is a variant of trauma-focused CBT, and that interventions based on CBT included in this review might have a trauma-focused component; therefore, there might be a varying degree of overlap in content between NET and CBT as provided in the included studies. Indirectly, this is also supported by very similar overall effect size estimates for NET and CBT studies.

The review has some limitations. A limited number of studies were included, with a relatively low total number of patients contributing to the primary analysis. It should be acknowledged, however, that conducting research with refugees and asylum seekers is challenging, as potential participants may have limited command of the language of the host country, which complicates psychological treatments and obtaining informed consent for participating in research. Moreover, potential participants may not fully understand the health care system, the rules governing research and the reasons for being offered both a psychosocial intervention and participation in a research trial. It would have been possible to follow a narrative rather than quantitative description of the evidence base, but we argue that the pooling process had several merits, including the possibility of detecting and quantifying between-study heterogeneity, which was then further investigated by means of subgroup analyses, and that of increasing the power of some comparisons. Another limitation is that the included studies differed with respect to country of origin, time since resettlement, year and country of study publication, outcome measures, content and modalities of delivering psychosocial interventions. This may explain statistical heterogeneity when all studies were pooled together. However, heterogeneity was decreased in some subgroup analyses. For example, the effectiveness of NET was supported by five RCTs showing a relatively large effect with reasonably low heterogeneity. In addition, we were not able to conduct subgroup analyses to investigate the role of some clinically important variables, such as the time since resettlement, as this information was not systematically reported, and the modality of delivering the intervention (individual versus group), as only one study on CROP and one on TFP used a group treatment format. We also cannot rule out the possibility that publication bias might have inflated the overall magnitude of treatment effect.

In terms of implications for practice and research, this review summarises the effectiveness of psychosocial interventions for PTSD in refugees in the context of high-income countries, and therefore provides an evidence base to inform decisions in clinical practice and in policy making. Overall, the review may be used to underpin calls for the provision of psychosocial treatments for refugees with PTSD, particularly CBT treatments with a trauma focus. This is also the case for refugees with PTSD and comorbid disorders, which is frequently reported. Yet, the provision of psychosocial interventions requires resources for the organisation of treatments, while refugees are in challenging contexts, as well as the staff time needed for the treatment sessions, training and supervision. Whether the expected benefits of psychosocial interventions outweigh the associated costs may depend on context factors and local standards of routine mental health care for refugees and asylum seekers. Furthermore, there is consensus amongst humanitarian agencies that specialized treatments for conflict-affected populations need to be integrated in a multi-layered and multi-sectoral system of care, which caters for the diverse mental health needs of refugees—including but not limited to PTSD [52–56].

The current evidence base needs to be expanded with more rigorous trials focusing on a wider range of mental health disorders. New studies will be essential to address several clinical aspects that have not yet been investigated. This includes longer-term studies to establish whether benefits are maintained over the time or even only materialise after some time, and comparative studies to test whether the effectiveness of psychosocial treatments differs between different groups, as treating newly arrived refugees might be different from treating already resettled people. Future research should also explore how interventions might be adapted based on refugees’ understanding of traumatic experiences and psychosocial distress. Finally, head-to-head studies may assess the comparative cost-effectiveness of different psychosocial interventions.

## Supporting information

S1 Table PRISMA checklist. (DOCX)

S2 Table Search strategy. (DOCX)

S3 Table Definitions. (DOCX)

S4 Table References to excluded studies with reasons. (DOCX)

S5 Table Description of psychosocial interventions. (DOCX)

S6 Table Risk of bias: review authors' judgements about each risk of bias item for each included study. (DOCX)

S7 Table GRADE summary of findings table. (DOCX)

S1 Fig Publication bias. (DOCX)

S2 Fig Impact of each study on the pooled effect (sensitivity analysis): consecutively removal of each study as a possible outlier to test what the impact is on the combined effect. (DOCX)

S3 Fig Forest plot of secondary outcomes not reported in the main report. (DOCX)

S4 Fig Forest plot of subgroup analysis–PTSD symptoms. (DOCX)

S5 Fig Unrestricted maximum likelihood random effects meta-regression analysis investigating the association between number of sessions and effect size (Hedges’s g). (DOCX)

S6 Fig Unrestricted maximum likelihood random effects meta-regression analysis investigating the association between length of follow-up and effect size (Hedges’s g). (DOCX)

S7 Fig Forest plot of subgroup analysis–Depressive symptoms. (DOCX)
