# Supplementary material for: Psychosocial interventions for post-traumatic stress disorder in refugees and asylum seekers resettled in high-income countries: Systematic review and meta-analysis
Source: PLoS One. 2017 Feb 2;12(2):e0171030. doi: 10.1371/journal.pone.0171030 (PMC5289495; doi:10.1371/journal.pone.0171030)
Supplement: S2 Table — (DOCX) [file pone.0171030.s002.docx]

# S2 Table. Search strategy

Two review authors (MN and FB) independently screened titles and abstracts for inclusion. Studies rated as possible candidates by either of the two reviewers were added to a preliminary list and their full texts were retrieved. Working independently and in duplicate, the two review authors inspected the full texts for inclusion. Following Cochrane methodology, disagreements were resolved through discussion. The search strategy reported below was employed.

- [AND in builder](http://www.ncbi.nlm.nih.gov/pubmed/advanced)
- [OR in builder](http://www.ncbi.nlm.nih.gov/pubmed/advanced)
- [NOT in builder](http://www.ncbi.nlm.nih.gov/pubmed/advanced)
- [Delete from history](http://www.ncbi.nlm.nih.gov/pubmed/advanced)
- [Show search results](http://www.ncbi.nlm.nih.gov/pubmed/advanced)
- [Show search details](http://www.ncbi.nlm.nih.gov/pubmed/advanced)
- [AND in builder](http://www.ncbi.nlm.nih.gov/pubmed/advanced)
- [OR in builder](http://www.ncbi.nlm.nih.gov/pubmed/advanced)
- [NOT in builder](http://www.ncbi.nlm.nih.gov/pubmed/advanced)
- [Delete from history](http://www.ncbi.nlm.nih.gov/pubmed/advanced)
- [Show search results](http://www.ncbi.nlm.nih.gov/pubmed/advanced)
- [Show search details](http://www.ncbi.nlm.nih.gov/pubmed/advanced)
- [Save in My NCBI](http://www.ncbi.nlm.nih.gov/pubmed/advanced)
- [AND in builder](http://www.ncbi.nlm.nih.gov/pubmed/advanced)
- [OR in builder](http://www.ncbi.nlm.nih.gov/pubmed/advanced)
- [NOT in builder](http://www.ncbi.nlm.nih.gov/pubmed/advanced)
- [Show search results](http://www.ncbi.nlm.nih.gov/pubmed/advanced)
- [Save as a My NCBI Collection](http://www.ncbi.nlm.nih.gov/pubmed/advanced)

| **PUBMED/MEDLINE** | |
| --- | --- |
| Search trial or intervention or randomis* or randomiz* or treatment or pharmacolog* or psychother* or psychological or psychosocial | [11148569](http://www.ncbi.nlm.nih.gov/pubmed/?cmd=HistorySearch&querykey=1) |
| Search PTSD or post traumatic stress disorder | [28867](http://www.ncbi.nlm.nih.gov/pubmed/?cmd=HistorySearch&querykey=2) |
| Search refugee OR asylum OR migrant OR displaced OR IDP OR torture | [53425](http://www.ncbi.nlm.nih.gov/pubmed/?cmd=HistorySearch&querykey=3) |
| Search ((refugee OR asylum OR migrant OR displaced OR IDP OR torture) AND (PTSD or post traumatic stress disorder)) AND (trial or intervention or randomis* or randomiz* or treatment or pharmacolog* or psychother* or psychological or psychosocial) | [**1115**](http://www.ncbi.nlm.nih.gov/pubmed/?cmd=HistorySearch&querykey=4) |
|  | |
| **PsycINFO** | |
| ab(trial or intervention or randomis* or randomiz* or treatment or pharmacolog* or psychother* or psychological or psychosocial) AND ab(PTSD or post traumatic stress disorder) AND ab(refugee OR asylum OR migrant OR displaced OR IDP OR torture) | **571** |
| **CINHAL** | |
| AB (trial or intervention or randomis* or randomiz* or treatment or pharmacolog* or psychother* or psychological or psychosocial) AND AB (PTSD or post traumatic stress disorder) AND AB (refugee OR asylum OR migrant OR displaced OR IDP OR torture) | **86** |
| **PILOTS** |  |
| (trial OR intervention OR randomise* OR randomize* OR treatment OR pharmacology* OR psychother* OR psychological OR psychosocial) AND ((refugee OR asylum OR migrant OR displaced OR IDP OR torture) AND (PTSD OR post traumatic stress disorder)) | **2207** |
| **CENTRAL** |  |
| ((refugee OR asylum OR migrant OR displaced OR IDP OR torture)) AND (PTSD or post traumatic stress disorder)) AND (trial or intervention or randomis* or randomiz* or treatment or pharmacolog* or psychother* or psychological or psychosocial) | **52** |
| **Web of Science** | |
| TOPIC: (trial or intervention or randomis* or randomiz* or treatment or pharmacolog* or psychother* or psychological or psychosocial) *AND* TOPIC: (PTSD or post traumatic stress disorder) *AND* TOPIC: (refugee OR asylum OR migrant OR displaced OR IDP OR torture) | **705** |
| **EMBASE** | |
| trial OR intervention OR randomis* OR randomiz* OR treatment OR pharmacolog* OR psychother* OR psychological OR psychosocial AND ('ptsd'/exp OR ptsd OR post) AND traumatic AND ('stress'/exp OR stress) AND ('disorder'/exp OR disorder) AND ('refugee'/exp OR refugee OR asylum OR 'migrant'/exp OR migrant OR displaced OR idp OR 'torture'/exp OR torture) | **654** |
| TOTAL search results  TOTAL search results after removing duplicates | **5390**  **3139** |
