# Supplementary material for: Psychosocial interventions for post-traumatic stress disorder in refugees and asylum seekers resettled in high-income countries: Systematic review and meta-analysis
Source: PLoS One. 2017 Feb 2;12(2):e0171030. doi: 10.1371/journal.pone.0171030 (PMC5289495; doi:10.1371/journal.pone.0171030)
Supplement: S3 Table — (DOCX) [file pone.0171030.s003.docx]

# S3 Table. Definitions

| PSYCHOSOCIAL INTERVENTION. Psychosocial intervention was defined as any type of therapy, education, training, or social support aimed at improving PTSD symptoms, behaviour, general functioning, or any other clinically relevant PTSD outcome dimension without the use of psychopharmacologic agents (1;2).1,2  ASYLUM SEEKER AND REFUGEE. Asylum seekers were defined, according to the 1951 United Nations Convention, as persons who have fled their own country and formally apply to the government of another country for asylum but the application has not yet been concluded; they remain asylum-seekers while they are awaiting a decision on their application for refugee status. People move from asylum seeker status to refugee status once the country they have applied for asylum in accepts their claim.  HIGH-INCOME COUNTRY. Countries were defined as “high-income” according to the World Bank criteria (3).3  References  (1) Tol WA, Barbui C, Galappatti A, Silove D, Betancourt TS, Souza R et al. Mental health and psychosocial support in humanitarian settings: linking practice and research. Lancet 2011; 378: 1581-91.  (2) Tol WA, Purgato M, Bass JK, Galappatti A, Eaton W. Mental health and psychosocial support in humanitarian settings: a public mental health perspective. Epidemiol Psychiatr Sci 2015; 24: 484-94.  (3) World Bank. The World Bank. Working for a world free of poverty. Available from: http://www worldbank org/ |
| --- |
