# Supplementary material for: Psychosocial interventions for post-traumatic stress disorder in refugees and asylum seekers resettled in high-income countries: Systematic review and meta-analysis
Source: PLoS One. 2017 Feb 2;12(2):e0171030. doi: 10.1371/journal.pone.0171030 (PMC5289495; doi:10.1371/journal.pone.0171030)
Supplement: S4 Table — (DOCX) [file pone.0171030.s004.docx]

# S4 Table. References to excluded studies with reasons

| **EXCLUDED STUDY** | **REASON** |
| --- | --- |
| 1. Acarturk C, Konuk E, Cetinkaya M, Senay I, Sijbrandij M, Cuijpers P, Aker T (2015). EMDR for Syrian refugees with posttraumatic stress disorder symptoms: results of a pilot randomized controlled trial. *European Journal of Psychotraumatology* 6:27414. | No High Income Country |
| 1. Arntz A, Sofi D, van Breukelen G (2013). Imagery Rescripting as treatment for complicated PTSD in refugees: a multiple baseline case series study. *Behaviour Research and Therapy* 51(6): 274-83 | No comparison group |
| 1. Berkson SY, Tor S, Mollica R, Lavelle J, Cosenza C (2014). An innovative model of culturally tailored health promotion groups for Cambodian survivors of torture. Torture. 24(1):1-16. | No comparison group |
| 1. Bichescu D(1), Neuner F, Schauer M, Elbert T. (2007) Narrative exposure therapy for political imprisonment-related chronic posttraumatic stress disorder and depression. *Behaviour Research and Therapy* 45(9):2212-20. | No refugee and no comparison group |
| 1. Birman D, Beehler S, Harris EM, Everson ML, Batia K, Liautaud J, Frazier S, Atkins M, Blanton S, Buwalda J, Fogg L, Cappella E. (2008) International Family, Adult, and Child Enhancement Services (FACES): a community-based comprehensive services model for refugee children in resettlement. *American Journal of Orthopsychiatry* 78(1):121-32. | Sample <18 years |
| 1. Bolton P, Lee C, Haroz EE, Murray L, Dorsey S, Robinson C, Ugueto AM, Bass J (2014). A transdiagnostic community-based mental health treatment for comorbid disorders: development and outcomes of a randomized controlled trial among burmese refugees in Thailand. *PLoS Medicine;* 11 (11): e1001757 | No High Income Countries |
| 1. Böttche M, Kuwert P, Pietrzak RH, Knaevelsrud C (2015). Predictors of outcome of an Internet-based cognitive-behavioural therapy for post-traumatic stress disorder in older adults. *Psychology and Psychotherapy* | No refugee people |
| 1. Buhmann C, Andersen I, Mortensen EL, Ryberg J, Nordentoft M, Ekstrøm M. (2015). Cognitive behavioral psychotherapeutic treatment at a psychiatric trauma clinic for refugees: description and evaluation. *Torture* 25(1):17-32. | No control group |
| 1. Buhmann C, Mortensen EL, Nordentoft M, Ryberg J, Ekstrøm M (2015). Follow-up study of the treatment outcomes at a psychiatric trauma clinic for refugees. *Torture* 25(1):1-16. | No control group |
| 1. Carlsson JM, Olsen DR, Kastrup M, Mortensen EL (2010). Late mental health changes in tortured refugees in multidisciplinary treatment. *The journal of nervous and mental disease*198(11):824-8. | No control group |
| 1. Cavaljuga S, Licanin I, Mulabegović N, Potkonjak D (2003).Therapeutic effects of two antidepressant agents in the treatment of posttraumatic stress disorder (PTSD). Bosn J Basic Med Sci 3(2):12-6. | No refugee people |
| 1. d'Ardenne P, Ruaro L, Cestari L, Fakhoury W, Priebe S (2007) Does interpreter-mediated CBT with traumatized refugee people work? A comparison of patient outcomes in East London. *Behav Cogn Psychother.* 35:293–301 | No control group |
| 1. Drožđek B, Kamperman AM, Bolwerk N, Tol WA, Kleber RJ (2012). Group therapy with male asylum seekers and refugees with posttraumatic stress disorder: a controlled comparison cohort study of three day-treatment programs. *The Journal of Nervous and Mental Disease* 200 (9):758-65. | Same sample of Drozdek et al, 2010 |
| 1. Drožđek B, Kamperman AM, Tol WA, Knipscheer JW, Kleber RJ (2013). Is legal status impacting outcomes of group therapy for posttraumatic stress disorder with male asylum seekers and refugees from Iran and Afghanistan? *BMC Psychiatry* 13:148. | No control group |
| 1. Drožđek B, Kamperman AM, Tol WA, Knipscheer JW, Kleber RJ (2014). Seven-year follow-up study of symptoms in asylum seekers and refugees with PTSD treated with trauma-focused groups. *Journal of Clinical Psychology* 70(4):376-87 | No control group |
| 1. Durà-Vilà G, Klasen H, Makatini Z, Rahimi Z, Hodes M (2013). Mental health problems of young refugees: duration of settlement, risk factors and community-based interventions. *Clinical Child Psychology and Psychiatry* 18(4): 604-23 | Sample <18 years |
| 1. Dybdahl R (2001). Children and mothers in war: an outcome study of a psychosocial intervention program. *Child Development* *72*(4), 1214-1230. | No High Income Countries |
| 1. Ellis BH, Miller AB, Abdi S, Barrett C, Blood EA, Betancourt TS (2013) Multi-tier mental health program for refugee youth. *Journal of consulting and clinical psychology* 81(1):129-40. | Sample <18 years |
| 1. Fox SH (2003). The Mandinka nosological system in the context of post-trauma syndromes. *Transcultural Psychiatry* *40*(4), 488-506. | No High Income Countries |
| 1. Goldsmith RE(1), Martin CG, Smith CP (2014). Systemic trauma. *Journal of Trauma & Dissociation* 15(2):117-32. | NO RCT or CCT |
| 1. Goodkind JR, Hess JM, Isakson B, LaNoue M, Githinji A, Roche N, Vadnais K, Parker DP (2014). Reducing Refugee Mental Health Disparities: A Community-Based Intervention to Address Post-Migration Stressors With African Adults. *Psychological Services*, 11(3), 333–346. | No control group |
| 1. Halvorsen JØ &Stenmark H (2010). Narrative exposure therapy for posttraumatic stress disorder in tortured refugees: a preliminary uncontrolled trial. S*candinavian Journal of Psychology* 51(6):495-502. | No control group |
| 1. Halvorsen JØ, Stenmark H, Neuner F, Nordahl HM (2014). Does dissociation moderate treatment outcomes of narrative exposure treatment for PTSD? A secondary analysis from a randomized controlled clinical trial. *Behaviour Research and Therapy*; 57:21–8. | Same sample of Stenmark et al., 2013 |
| 1. Hárdi, L., & Kroo, A. (2011). Psychotherapy and psychosocial care of torture survivor refugees in Hungary. *Torture Volume*, (21), 84-97. | No control group |
| 1. Heavey, E. (2014). Female refugees: Sensitive care needed. Nursing2015, 44(5), 28-34. | NO RCT or CCT |
| 1. Hensel-Dittmann D, Schauer M, Ruf M, Catani C, Odenwald M, Elbert T, Neuner F (2011). Treatment of traumatized victims of war and torture: a randomized controlled comparison of narrative exposure therapy and stress inoculation training. *Psychotherapy and Psychosomatics*; 80(6):345–52. | Active comparator |
| 1. Hinton DE, Hofmann SG, Pollack MH, Otto MW (2009) Mechanisms of efficacy of CBT for Cambodian refugees with PTSD: improvement in emotion regulation and orthostatic blood pressure response. *CNS neuro science & therapeutics* 15(3): 255-63. | No control group |
| 1. Hinton DE, Kredlow MA, Bui E, Pollack MH, Hofmann SG (2012). Treatment change of somatic symptoms and cultural syndromes among Cambodian refugees with PTSD. *Depression and Anxiety.* 29(2):147-54. | No control group |
| 1. Hinton DE, Hofmann SG, Rivera E, Otto MW, Pollack MH (2011). Culturally adapted CBT (CA-CBT) for Latino women with treatment-resistant PTSD: a pilot study comparing CA-CBT to applied muscle relaxation. *Behaviour Research and Therapy* 49(4):275-80. | No refugee people |
| 1. Hussain D, Bhushan B (2011). Posttraumatic stress and growth among Tibetan refugees: the mediating role of cognitive‐emotional regulation strategies. *Journal of clinical psychology* *67*(7), 720-735. | No High Income Countries |
| 1. Jespersen KV, Vuust P (2012). The Effect of Relaxation Music Listening on Sleep Quality in Traumatized Refugees: A Pilot Study. *J Music Ther.* 49(2):205-29. | PTSD is not primary outcome |
| 1. Keller AS(1), Saul JM, Eisenman DP (1998). Caring for survivors of torture in an urban, municipal hospital. J Ambul Care Manage. 1998 Apr;21(2):20-9; discussion 43-55. | No RCT or CCT |
| 1. Kinzie JD(1), Kinzie JM, Sedighi B, Woticha A, Mohamed H, Riley C (2012). Prospective one-year treatment outcomes of tortured refugees: a psychiatric approach. *Torture* 22(1): 1-10. | No control group |
| 1. Kira IA, Ahmed A, Mahmoud V, Wasim F.(2010) Group therapy model for refugee and torture survivors. *Torture* 20(2):108-13. | NO RCT or CCT |
| 1. Knaevelsrud C &Maercker A (2006).Does the quality of the working alliance predict treatment outcome in online psychotherapy for traumatized patients?*Journal of medical internet research*8(4):e31. | No refugee people |
| 1. Knaevelsrud C(1), Liedl A, Maercker A (2010). Posttraumatic growth, optimism and openness as outcomes of a cognitive-behavioural intervention for posttraumatic stress reactions. *Journal of health psychology* 15(7):1030-8. | No refugee people |
| 1. Knaevelsrud C(1), Wagner B, Karl A, Mueller J (2007). New treatment approaches: integrating new media in the treatment of war and torture victims. *Torture* 2007;17(2):67-78. | No control group (description of the intervention) |
| 1. Knaevelsrud C, & Maercker A (2007). Internet-based treatment for PTSD reduces distress and facilitates the development of a strong therapeutic alliance: a randomized controlled clinical trial. *BMC Psychiatry* 7, 13. | No refugee people |
| 1. Kulwicki, A., & Ballout, S. (2015). Post Traumatic Stress Disorder (PTSD) in Arab American refugee and recent immigrant women. *Journal of Cultural Diversity*, *22*(1). | NO RCT or CCT |
| 1. Manneschmidt S & Griese K (2009). Evaluating psychosocial group counselling with afghan women: is this a useful intervention? *Torture* 19(1): 41-50. | No High Income Countries |
| 1. Mirdal GM, Ryding E, EssendropSondej M (2012). Traumatized refugees, their therapists, and their interpreters: three perspectives on psychological treatment. *Psychology and Psychotherapy* 85(4): 436-55. | NO RCT or CCT |
| 1. Mirdal GM, Ryding E, EssendropSondej M (2012). Traumatized refugees, their therapists, and their interpreters: three perspectives on psychological treatment. *Psychology and Psychotherapy* 85(4): 436-55. | NO RCT or CCT |
| 1. Muller J, Karl A, Denke C, Mathier F, Dittmann J, Rohleder N, Knaevelsrud C (2009). Biofeedback for pain management in traumatised refugees. *Cognitive behaviour therapy* 38(3):184-90. | No control group |
| 1. Palic S, Elklit A (2009). An explorative outcome study of CBT-based multidisciplinary treatment in a diverse group of refugees from a Danish treatment centre for rehabilitation of traumatized refugees. *Torture* 19(3):248-70. | No control group |
| 1. Paunovic N, Öst LG (2001). Cognitive-behavior therapy vs. exposure therapy in the treatment of PTSD in refugees. *Behaviour Research and Therapy* 39: 1183–1197. | Active comparator |
| 1. Pokhariyal G P, Rono R, Munywoki S (2012). Analysis of Treatment Methods for Victims of Torture in Kenya and East Africa Region. *Traumatology* 19(2) 107– 117 | No High Income Countries |
| 1. Premand N, Baeriswyl-Cottin R, Gex-Fabry M, Coraboeuf B, Giannakopoulos P, Eytan A, Bartolomei J (2013). Psychiatric care for asylum seekers in Geneva: a multidisciplinary approach for individualized care. *Revue Médicale Suisse* 9(398): 1664-8. | NO RCT or CCT |
| 1. Renner W (2009). The effectiveness of psychotherapy with refugees and asylum seekers: preliminary results from an Austrian study. *Journal of Immigrant and Minority Health* 11(1):41-5. | No control group |
| 1. Robertson ME, Blumberg JM, Gratton JL, Walsh EG, Kayal H(2013). A group-based approach to stabilisation and symptom management in a phased treatment model for refugees and asylum seekers. *European Journal of Psychotraumatology* 20;4 | NO RCT or CCT(descriptive paper) |
| 1. Schaal S, Elbert T, Neuner F (2009). Narrative exposure therapy versus interpersonal psychotherapy. *Psychotherapy and Psychosomatics* *78*(5), 298-306. | No High Income Countries |
| 1. Schauer M, Elbert T, Gotthardt S, Rockstroh B, Odenwald M, Neuner F (2006). Imaginary reliving in psychotherapy modifies mind and brain [Wiedererfahrung durch Psychotherapie modifiziert Geistund Gehirn*]. *Verhaltenstherapie*;16 (2):96–103. | NO RCT or CCT |
| 1. Schulz PM, Resick PA, Huber LC, Griffin MG (2006) The effectiveness of cognitive processing therapy for PTSD with refugees in a community setting. *Cogn Behav Pract*. 13:322–331. | No control group |
| 1. Schweitzer R(1), Melville F, Steel Z, Lacherez P (2006). Trauma, post-migration living difficulties, and social support as predictors of psychological adjustment in resettled Sudanese refugees. *Australian and New Zealand journal of psychiatry*40(2):179-87. | NO RCT or CCT |
| 1. Silove D, Manicavasagar V, Beltran R, Le G, Nguyen H, Phan T, Blaszczynski A (1997). Satisfaction of Vietnamese patients and their families with refugee and mainstream mental health services. *Psychiatric Services* 48(8):1064-9. | No control group |
| 1. Slewa-Younan S, Mond J, Bussion E, Mohammad Y, Uribe Guajardo MG, Smith M, Milosevic D, Lujic S, Jorm AF (2014). Mental health literacy of resettled Iraqi refugees in Australia: knowledge about posttraumatic stress disorder and beliefs about helpfulness of interventions. *BMC Psychiatry*. 14:320 | No control group |
| 1. Smajkic A, Weine S, Djuric-Bijedic Z, Boskailo E, Lewis J, & Pavkovicl I (2001). Sertraline, paroxetine, and venlafaxine in refugee posttraumatic stress disorder with depression symptoms. *Journal of Traumatic Stress;* 14(3): 445–452. | Comparison among pharmacological interventions |
| 1. Smid GE, Kleber R J, Simone M, Bos JB, Gersons BP, Boelen PA (2015). Brief Eclectic Psychotherapy for Traumatic Grief (BEP-TG): toward integrated treatment of symptoms related to traumatic loss. *European journal of psychotraumatology*, *6*. | NO RCT or CCT |
| 1. Sonne C, Carlsson J, Elklit A, Mortensen EL, Ekstrøm M (2013). Treatment of traumatized refugees with sertraline versus venlafaxine in combination with psychotherapy-study protocol for a randomized clinical trial. *Trials*; 14:137. | NO RCT or CCT (Protocol) |
| 1. Stenmark H, Guzey IC, Elbert T, Holen A(2014). Gender and offender status predicting treatment success in refugees and asylum seekers with PTSD. *European Journal of Psychotraumatology* 30;5. | No control group |
| 1. Strasser J, Chhim S, & Taing S (2012). Narrative Exposure Therapy (NET): Culturally Sensitive Trauma Treatment for Khmer Rouge Survivors. *The International Association of Buddhist Universities (IABU)*, 100 | No refugee people |
| 1. Ter Heide FJ, Mooren TM, van de Schoot R, de Jongh A, Kleber RJ (2016).Eye movement desensitisation and reprocessing therapy v. stabilisation as usual for refugees: randomised controlled trial.*The British Journal of Psychiatry*  bjp.bp.115.167775. [Epub ahead of print] | Active comparator |
| 1. ter Heide JJ, Mooren TM, Kleijn W, de Jongh A, Kleber RJ (2011). EMDR versus stabilisation in traumatised asylum seekers and refugees: results of a pilot study. *European Journal of Psychotraumatology* 2: 5881. | Active comparator |
| 1. van Melle MA, Lamkaddem M, Stuiver MM, Gerritsen AA, Devillé WL, Essink-Bot ML (2014). Quality of primary care for resettled refugees in the Netherlands with chronic mental and physical health problems: a cross-sectional analysis of medical records and interview data. *BMC Family* 15:160. | No control group |
| 1. Vindbjerg E, Klimpke C, Carlsson J (2014). Psychotherapy with traumatised refugees--the design of a randomised clinical trial. *Torture* 24(1):40-8. | NO RCT or CCT (Protocol) |
| 1. Wagner B, Knaevelsrud C, Maercker A (2006). Internet-based cognitive- behavioral therapy for complicated grief: a randomized controlled trial. *Death Studies* 30(5):429-53. | No refugee people |
