# Supplementary material for: Psychosocial interventions for post-traumatic stress disorder in refugees and asylum seekers resettled in high-income countries: Systematic review and meta-analysis
Source: PLoS One. 2017 Feb 2;12(2):e0171030. doi: 10.1371/journal.pone.0171030 (PMC5289495; doi:10.1371/journal.pone.0171030)
Supplement: S5 Table — (DOCX) [file pone.0171030.s005.docx]

# S5 Table. Description of psychosocial interventions

| Study | Intervention | Sessions (n° and length) | Intervention Agent, Training, and Fidelity |
| --- | --- | --- | --- |
| Adenauer 2011 | Narrative Exposure Therapy (NET): manualized short-term variant of trauma-focused CBT, adapted to meet the needs of traumatized survivors  of war and torture. During the therapy sessions, the patient, assisted by the therapist, constructs a detailed chronological account of his or her own biography. The autobiography is recorded by the therapist in written form and is corrected and elaborated on each subsequent reading. The therapist writes down the biography and reads it aloud at the beginning of each following session for completion and correction. The aim of the therapy is the reorganization of the generally fragmented report of traumatic experiences into a coherent narrative. During the confrontation with the aversive life events, the therapist asks for current and past emotional, physiological, cognitive, and behavioural reactions, and probes for respective observations. During the last session, the participant receives the written report of the biography. In order to meet the needs of patients with insecure asylum status, the last two sessions were kept flexible. | 12 weekly sessions, lasting on average 108 minutes | Clinical psychologists with expertise in PTSD and NET with the help of a translator if necessary. Regular supervision; all testimonies written down during NET treatment by the therapists were checked for indicators of vividness and consistency to ensure proper application of NET |
| Buhmann 2016 | Cognitive Behavior Treatment (CBT): manualized treatment developed in cooperation with specialists in CBT, including core CBT methods, methods from acceptance and commitment therapy (ACT), mindfulness exercises and in vivo and visualised exposure. | 16 sessions | Psychologists trained in CBT; supervision by specialists in CBT. The use of the various methods in the manual was logged on a standard checklist so that a fidelity check could be made in the analysis. To determine adherence, psychoeducation topics covered, psychotherapeutic methods used and cooperation with medical treatment were registered at each session. |
| Drozdek 2010 | Trauma-focused psychotherapy: treatment model for asylum seekers and refugees, known as the Den Bosch treatment model. It consists of a phase-oriented trauma treatment based on an adapted five-phase model. The treatment combines psychodynamic, cognitive-behavioural, and supportive approaches and is designed to help asylum seekers and refugees work through their traumatic experiences and place them in a life-span developmental perspective.  . | 85 biweekly group sessions lasting 90 minutes, combined with 2 or 3 different nonverbal therapy sessions lasting 75 minutes in the same week. | - |
| Hijazi 2014 | Brief Narrative Exposure Therapy (NET): manualised intervention adapted. Session 1 started with psychoeducation, including normalization of the participant’s experience and explanation of the therapy rationale. The participant then constructed a chronological narrative of his or her life, starting with highlights of childhood and then focusing on traumatic experiences during adulthood. While the participant was narrating, the therapist handwrote the story and later transcribed it on a computer (in Arabic) before the next session. In Session 2, the therapist read aloud the narrative, which the participant revised or enhanced. The process of constructing the life narrative continued, again focusing on processing traumatic experiences. Session 3 involved re-reading the participant’s narrative and discussing his or her fears, goals, and hopes for the future. A paper copy of the narrative was provided to the participant at the session’s end. | 3 sessions, lasting 60–90 minutes. | Therapists received training and weekly supervision by a licensed psychologist with expertise in exposure therapies. |
| Hinton 2004 | Cognitive Behavior Treatment (CBT): utilizing a manual-based protocol developed by the first author. During CBT, the therapist stressed 10 core elements: 1. providing information about the nature of PTSD and panic disorder such as how trauma reminders and catastrophic cognitions generate panic attacks; 2. training in muscle relaxation and diaphragmatic breathing procedures, including those of applied relaxation techniques; 3. instruction in a culturally appropriate visualization and having the patient perform analogous rotational movements at the neck after each relaxation of the neck and head musculature; 4. framing relaxation techniques as a form of mindfulness, that is, as an attentive focusing upon specific sensory; 5. cognitive restructuring of fear networks, especially trauma memories and catastrophic misinterpretations of somatic sensations  6. conducting interoceptive exposure to anxiety-related sensations to treat panic attacks generated by catastrophic cognitions, trauma associations, and interoceptive conditioning to those sensations; 7. providing an emotional processing protocol to utilize during times of trauma recall, the protocol bringing about a shift from an attitude of pained acceptance to one of mindfulness; 8. exploring headache and orthostatic panic, as in investigating firing sequences—e.g. the sensations, activities, and thoughts that initiate the sequence leading to panic—and associated catastrophic cognitions and trauma associations. | 11 weekly sessions | - |
| Hinton 2005 | Cognitive Behavior Treatment (CBT): utilizing a manual-based protocol developed by the first author. See Hinton 2004. | 12 weekly sessions | - |
| Kruse 2009 | Trauma-focused psychotherapy: manualised psychotherapy (adapted the first phase of consensus model of trauma therapy), focusing on skills training in affective regulation and interpersonal relationships with the goals of symptom stabilization and improved all-day functioning. | 25 hours once a week (1-3 months) then every 2 weeks; sessions lasting 50 minutes | Psychotherapist with 5 years of training in psychotherapy (CBT and psychodynamic) and also trained in trauma specific psychotherapy. |
| Liedl 2011 | Cognitive Behavior Treatment (CBT)-Biofeed back (BF) intervention: in the initial session, therapist and patient discussed predominant physical and psychological problems as well as problems of the patient’s current life situation. Psychoeducation was then provided about the relationship between stress and pain. In the second session, therapist and patient jointly developed an individual formulation of the patient’s current symptoms and possible routes to recovery. The treatment procedure and goals were discussed, with the stated rationale for the procedure being adapted to the patient’s educational level and/or level of comprehension. In sessions 3 through 9, therapist and patient focused on the patient’s primary pain. Various relaxation strategies were introduced and trained with the help of CBT-BF. The BF sessions consisted of the following phases: baseline, stress situation, pain situation), relaxation situation and muscle relaxation and adaptation phases. In session 9, pain-related problems such as dysfunctional cognitions and behaviour or inactivity were modified using standard CBT techniques. In session 10, the methods and strategies that patients had learned to cope with chronic pain were reviewed and evaluated. In addition, patients were instructed to practice progressive muscle relaxation at home. | 10 weekly  sessions, lasting 90  minutes | Graduate students in clinical psychology trained in the application of CBT-BF to treat pain in traumatized refugees and torture survivors and who had observed a professional CBT-BF therapist. The graduate students participated in weekly supervision sessions. Homework adherence was monitored in every session by asking participants about their experiences and problems of relaxation training at home. |
| Morath 2014 | Narrative Exposure Therapy (NET): manualized short-term variant of trauma-focused CBT, for PTSD, developed for survivors of war and torture. | 12 weekly sessions  lasting 90 min | Clinical psychologists specialized in the field of trauma and  experts for narrative exposure therapy (NET).  Treatment adherence was monitored by regular supervision. |
| Neuner 2010 | Narrative Exposure Therapy (NET): manualized short-term variant of trauma-focused CBT, for PTSD, developed for survivors of war and torture. The first session included psychoeducation about the nature of PTSD symptoms and the NET rationale. Treatment was terminated at the therapist’s discretion. As soon as the patient could, according to clinical judgment, talk about his or her traumatic experiences in detail without avoidance, memory gaps, or being emotionally overwhelmed, NET could be ended. In the last session, the participant received a written testimony of his or her biography. | 9 weekly or biweekly sessions lasting on average 120 minutes | Therapists were doctoral-level psychologists and graduate students with extensive training in NET. The authors supervised the treatment. In supervision meetings, treatment progress, including the records of life histories, was evaluated. In addition, randomly selected treatment sessions were directly observed by authors. Furthermore, all testimonies gained in NET treatment were checked for indicators of vividness and consistency to ensure proper application of NET. |
| Otto 2003 | Cognitive Behavior Treatment (CBT): CBT treatment focusing on the following elements: (1) information on the symptoms and nature of PTSD  from a cognitive-behavioural perspective; (2) clarification of the difference between PTSD symptoms and culturally-distinct fears of death or disability associated with somatic sensations; (3) exposure to somatic sensations associated with PTSD and anxiety; (4) exposure to memories of specific trauma events with rehearsal of emotional acceptance and cognitive coping strategies; (5) progressive muscle relaxation and diaphragmatic breathing skills, and (6) self-care skills and assignment of pleasant events. Treatment was conducted in a group format and was held in a local Buddhist temple. | 10 group sessions | - |
| Renner 2011 | Culture-Sensitive and Resource Oriented Peer Groups (CROP): self-help groups for refugees and asylum seeker, in the presence of facilitators, with the aim of reducing symptoms of posttraumatic stress including culturally specific elements of coping with trauma or distress. | 16 weekly group sessions lasting 90 minutes. | Trained people as CROP group facilitators with 180 hours of training. |
| Stenmark 2013 | Narrative Exposure Therapy (NET): manualized short-term variant of trauma-focused CBT, for PTSD, developed for survivors of war and torture. | 10 weekly sessions lasting 90 minutes. | Experienced mental health professionals received specific training in the assessments and in the NET (i.e., a total of five days followed by supervision). Subsequently, the therapists attended two-day supervision gatherings every six months to maintain their skills. Treatment adherence was monitored through supervision and through self-report measures after each session. |
| Weine 2008 | Coffee and Family Education and Support (CAFES) intervention: community-based, family-focused program that is aimed at improving access to mental health services by impacting family processes . It was facilitated by other Bosnian refugees who were laypersons trained and supervised by a university ⁄ community team. CAFES was based upon family strength and resilience approaches which emphasized the roles of family processes in facilitating adjustment, recovery, and development. The primary subject and all family members over age 17 were invited to attend. Each meeting began with casual conversation between families, and then the facilitators introduced a topic with a 15-minute didactic talk, which was followed by a 1-hr family discussion. | 9 multiple-family group sessions over 16 weeks | Trained female group facilitators, assigned to each multiple-family group. Intervention integrity was addressed by providing 20 hr of implementation training, weekly group and individual supervision, and monthly videotaping of the CAFES sessions, all by an experienced family therapist. |
|  |  |  |  |
