# Supplementary material for: Psychosocial interventions for post-traumatic stress disorder in refugees and asylum seekers resettled in high-income countries: Systematic review and meta-analysis
Source: PLoS One. 2017 Feb 2;12(2):e0171030. doi: 10.1371/journal.pone.0171030 (PMC5289495; doi:10.1371/journal.pone.0171030)
Supplement: S6 Table — (DOCX) [file pone.0171030.s006.docx]

# S6 Table. Risk of bias: review authors' judgements about each risk of bias item for each included study

##

## Adenauer 2011

| **Bias** | **Authors' judgement** | **Support for judgement** |
| --- | --- | --- |
| Random sequence generation (selection bias) | Low risk | quote: "participants... were randomised using a computer-generated list of random numbers" |
| Allocation concealment (selection bias) | Unclear risk | no details provided |
| Blinding of participants and personnel (performance bias) | High risk | participants not blind (waiting list); personnel cannot be blind for this type of treatment |
| Blinding of outcome assessment (detection bias) | Low risk | post test were carried out by interviewers who were blind to treatment condition |
| Incomplete outcome data (attrition bias) | Low risk | analysis carried out only on completers; however, the number of dropout is very low, balanced across intervention groups with similar reasons for missing data across groups, that are not related to the outcome (patients moved for deportation) |
| Selective reporting (reporting bias) | Low risk | all expected outcomes were clearly reported |
| Other bias | Unclear risk | two authors are authors of a NET manual |

## Buhmann 2016

| **Bias** | **Authors' judgement** | **Support for judgement** |
| --- | --- | --- |
| Random sequence generation (selection bias) | Low risk | QUOTE: "the randomisation sequence was computer generated by the Department of Biostatistics at University of Copenaghen, which was not otherwise involved in the research project. Randomisation was stratified by gender and total score on HTQ, so that patients with equal illness severity were allocated to all groups." |
| Allocation concealment (selection bias) | Low risk | QUOTE: "allocation was concealed by using sequentially numbered sealed envelopes. The envelopes were kept in an office physically separate from the clinic and were administered by secretaries who were not associated with the research project. When a patient had been included in the trial, the physician telephoned the office administering the randomisation envelopes and patients were subsequently assigned to a treatment group." |
| Blinding of participants and personnel (performance bias) | High risk | QUOTE: "it was not deemed possible to mask the patients, physicians, or psychologists to the treatment group because of the substantial differences between the treatment modalities" |
| Blinding of outcome assessment (detection bias) | Low risk | QUOTE: "a group of medical students not otherwise involved in the treatment undertook the masked ratings and met regularly to increase rater reliability" |
| Incomplete outcome data (attrition bias) | Unclear risk | missing data have been imputed using appropriated method (full information maximum likelihood- FIML); however number of patients included in the analyses corresponds to completers only. Data on drop out rates are unclear |
| Selective reporting (reporting bias) | Low risk | all expected outcomes were clearly reported |
| Other bias | Unclear risk | heterogeneity of participants in terms of culture and language; assessment tools not validated to the language and culture of participants; the trial was funded by the capital region of Copenaghen |

## Drozdek 2010

| **Bias** | **Authors' judgement** | **Support for judgement** |
| --- | --- | --- |
| Random sequence generation (selection bias) | High risk | the study is not randomised |
| Allocation concealment (selection bias) | High risk | see above |
| Blinding of participants and personnel (performance bias) | High risk | participants and personnel not blind |
| Blinding of outcome assessment (detection bias) | High risk | outcome assessment was not blind |
| Incomplete outcome data (attrition bias) | Unclear risk | QUOTE: "cases with missing data were removed from the analyses"; drop out were exceptional and they were excluded |
| Selective reporting (reporting bias) | Low risk | all outcomes were reported at baseline and at post intervention |
| Other bias | Unclear risk | all participants were Farsi and Dari speaking from Iran and Afghanistan; all measures were validated across cultural settings |

## Hijazi 2014

| **Bias** | **Authors' judgement** | **Support for judgement** |
| --- | --- | --- |
| Random sequence generation (selection bias) | Low risk | QUOTE: "the computerized scheme was stratified by recruitment site (agency) and assistance, and randomised the two conditions in blocks of six in a 2:1 ratio" |
| Allocation concealment (selection bias) | Low risk | QUOTE: "the assistant (heretofore blind to condition assignment) opened a sealed envelope and informed the participants when he or she would be getting the treatment" |
| Blinding of participants and personnel (performance bias) | High risk | participants and personnel not blind (see above) |
| Blinding of outcome assessment (detection bias) | High risk | measures were self-administered by the patients. QUOTE: "all participants were mailed follow up assessment measure and returned envelopes 2 and 4 months after measure" |
| Incomplete outcome data (attrition bias) | Low risk | QUOTE: "our primary analyses were intent-to-treat, meaning that we retained all 36 participants, regardless of how many intervention or follow-up assessment sessions they completed. Any missing follow-up data were replaced using the multiple imputation procedure in SPSS." |
| Selective reporting (reporting bias) | Low risk | all expected outcomes were clearly reported at all follow-up |
| Other bias | Unclear risk | the personnel were Arabic-speaking as the participants and the measures were translated into Arabic and most of the translated versions were validated |

## Hinton 2004

| **Bias** | **Authors' judgement** | **Support for judgement** |
| --- | --- | --- |
| Random sequence generation (selection bias) | Unclear risk | QUOTE: "the patients were randomly assigned to two cohorts of 6 each". No further details provided |
| Allocation concealment (selection bias) | Unclear risk | no information provided |
| Blinding of participants and personnel (performance bias) | High risk | participants not blind (waiting list); personnel cannot be blind for this type of treatment |
| Blinding of outcome assessment (detection bias) | High risk | measures were self-administered by the patients. QUOTE: "the participants completed the measures at three time points" |
| Incomplete outcome data (attrition bias) | Unclear risk | data at three endpoints are provided for all randomised patients. No details provided on drop-out and eventual methods to impute missing data |
| Selective reporting (reporting bias) | Low risk | all expected outcomes were clearly reported at all follow-up |
| Other bias | Unclear risk | one therapist led the CBT session and Vietnamese social workers and staff provided translation and cultural consultation; all patients were Vietnamese; the measures were translated and validated for Vietnamese population |

## Hinton 2005

| **Bias** | **Authors' judgement** | **Support for judgement** |
| --- | --- | --- |
| Random sequence generation (selection bias) | Low risk | QUOTE: "patients... were stratified by gender, with random allocation to either the Initial treatment, or the Delayed Treatment Groups decided by a coin toss" |
| Allocation concealment (selection bias) | Unclear risk | no information provided |
| Blinding of participants and personnel (performance bias) | High risk | participants not blind (waiting list); personnel cannot be blind for this type of treatment |
| Blinding of outcome assessment (detection bias) | Low risk | QUOTE: "blind to treatment condition, all assessments were made by a Cambodian bicultural worker" |
| Incomplete outcome data (attrition bias) | Low risk | data at three endpoints are provided for all randomised patients; all randomised patients completed the study and there were no missing data |
| Selective reporting (reporting bias) | Low risk | all expected outcomes were clearly reported at all follow-up |
| Other bias | Unclear risk | all patients were Cambodian and CBT session were conducted by one therapist who is fluent in Cambodian; all measures were translated and then back-translated |

## Kruse 2009

| **Bias** | **Authors' judgement** | **Support for judgement** |
| --- | --- | --- |
| Random sequence generation (selection bias) | High risk | the study is not randomised; QUOTE: "the first 35 patients were consecutively included in the treatment group, and the following 35 in the comparison group" |
| Allocation concealment (selection bias) | High risk | see above |
| Blinding of participants and personnel (performance bias) | High risk | participants not blind (waiting list); personnel cannot be blind for this type of treatment |
| Blinding of outcome assessment (detection bias) | High risk | QUOTE: "each patient filled out a questionnaire booklet" |
| Incomplete outcome data (attrition bias) | High risk | only completers data were analysed; numbers and reason for missing data across intervention groups were imbalanced; Reasons for missing data were reported (3 patients in the control group refused follow-up) |
| Selective reporting (reporting bias) | Low risk | all expected outcomes were clearly reported at all follow-up |
| Other bias | Unclear risk | all the participants were Bosnian; it is not clear if the measures were all translated into Bosnian language; the trial was funded by medical faculty of the University of Dusseldorf |

## Liedl 2011

| **Bias** | **Authors' judgement** | **Support for judgement** |
| --- | --- | --- |
| Random sequence generation (selection bias) | Unclear risk | QUOTE: "...were randomly assigned to one of the three conditions" |
| Allocation concealment (selection bias) | Unclear risk | no information provided |
| Blinding of participants and personnel (performance bias) | High risk | participants not blind (waiting list); personnel cannot be blind for this type of treatment |
| Blinding of outcome assessment (detection bias) | High risk | QUOTE: "the questionnaire were administered using multilingual computer assisted self interview..." |
| Incomplete outcome data (attrition bias) | Low risk | only completers data were analysed; missing outcome data were low and balanced in numbers across intervention groups, with similar reasons for missing data across groups |
| Selective reporting (reporting bias) | Low risk | all expected outcomes were clearly reported |
| Other bias | Unclear risk | patients were heterogeneous in terms of country of origin. QUOTE: "wherever possible, we used validated version of the questionnaire in the participants native languages". This paper was retracted for reasons not related to its scientific validity.  This article was retracted with this note:  The following article has been retracted by the authors due to irregularities they had found regarding compliance with study procedures and data management. Data files comprising details of participants that were not de-identified were sent between study sites. At the Zurich study site, research sessions and interpreter costs were partly charged to the insurance companies and the hospital rather than to the appropriate research account. Data quality, data analyses, and clinical conclusions drawn from the results were not affected. |

## Morath 2014

| **Bias** | **Authors' judgement** | **Support for judgement** |
| --- | --- | --- |
| Random sequence generation (selection bias) | Unclear risk | QUOTE: "refugees were randomly assigned to either a treatment (NET) or a wait list control condition" |
| Allocation concealment (selection bias) | Unclear risk | no information provided |
| Blinding of participants and personnel (performance bias) | High risk | participants not blind (waiting list); personnel cannot be blind for this type of treatment |
| Blinding of outcome assessment (detection bias) | Low risk | QUOTE: "the clinicians who performed the outcome evaluation were never the same as the clinicians who performed the baseline evaluation or the psychoterapeutic intervention; moreover the two follow-up evaluations were performed by different clinicians. Diagnosticians were blind with regard to group membership at baseline and at both post-tests" |
| Incomplete outcome data (attrition bias) | Low risk | analysis was conducted on all randomised patients. QUOTE: "with respect to missing values analyses were done using mixed model procedure" |
| Selective reporting (reporting bias) | Low risk | all expected outcomes were clearly reported at t1 (4 months) |
| Other bias | Unclear risk | QUOTE: "therapists were clinical psychologists specialised in the field of trauma and experts for NET. At baseline if participants were not fluent in English or German, diagnostic interviews were completed with the help of trained interpreters". Patients were heterogeneous in terms of country of origin.  QUOTE: "this study was funded by the German research foundation and European refugee fund. The funding source had no involvement in study design; in the collection, analyses and interpretation of data; in the writing of the report; and in the decision to submit the paper for publication". Two of the authors of the paper are authors of the NET manual |

## Neuner 2010

| **Bias** | **Authors' judgement** | **Support for judgement** |
| --- | --- | --- |
| Random sequence generation (selection bias) | Low risk | QUOTE: "participants were randomised into the two groups using a block permutation procedure with blocks of four patients" |
| Allocation concealment (selection bias) | Unclear risk | no information provided |
| Blinding of participants and personnel (performance bias) | High risk | personnel cannot be blind for this type of treatment. Participants in the control group "received the treatment, including psychotherapy and psychoactive medication. None of the patient in the TAU group received trauma focused treatment" |
| Blinding of outcome assessment (detection bias) | Low risk | QUOTE: "we aimed at keeping interviewers blind to each participant's condition. However, occasionally, the partecipants revealed their condition to the interviewer, despite instruction not to do so" |
| Incomplete outcome data (attrition bias) | Unclear risk | QUOTE: "we chose to apply mixed effects models that allow the inclusion all available data....". However results are reported at post treatment only for completers. Only two patients dropped out from the NET group, one for reasons related to the treatment |
| Selective reporting (reporting bias) | Low risk | all expected outcomes were clearly reported at endpoint |
| Other bias | Unclear risk | patients were heterogeneous in terms of country of origin.  QUOTE: "All instruments were assessed in the form of structured interviews. NET treatment was carried out according to the manual by therapists from the University of Konstanz with the help of trained interpreters".  Two of the authors of the paper are authors of the NET manual; Study was funded by European Refugee Fund |

## Otto 2003

| **Bias** | **Authors' judgement** | **Support for judgement** |
| --- | --- | --- |
| Random sequence generation (selection bias) | Unclear risk | QUOTE: "five patients were randomly assigned to sertraline treatment, and five to sertraline treatment plus ten sessions of CBT" |
| Allocation concealment (selection bias) | Unclear risk | no information provided |
| Blinding of participants and personnel (performance bias) | High risk | participants not blind; personnel cannot be blind for this type of treatment |
| Blinding of outcome assessment (detection bias) | Unclear risk | no information provided |
| Incomplete outcome data (attrition bias) | Unclear risk | drop-out data are not reported |
| Selective reporting (reporting bias) | Unclear risk | all expected outcomes were clearly reported, even if the total score of primay outcome is not reported |
| Other bias | Unclear risk | all participants were Cambodian (Khmer-speaking); treatment services were provided in Khmer; most of the scales have been validated for Khmer population |

## Renner 2011

| **Bias** | **Authors' judgement** | **Support for judgement** |
| --- | --- | --- |
| Random sequence generation (selection bias) | Unclear risk | QUOTE: "participants were assigned to the above mentioned conditions at random". Number of patients randomised for each group is not reported |
| Allocation concealment (selection bias) | Unclear risk | no information provided |
| Blinding of participants and personnel (performance bias) | High risk | participants not blind (waiting list). Personnel cannot be blind for this type of treatment |
| Blinding of outcome assessment (detection bias) | Unclear risk | no information provided |
| Incomplete outcome data (attrition bias) | High risk | QUOTE: "in all the groups there was a substantial drop out rate". Number of drop out for each group is not reported |
| Selective reporting (reporting bias) | High risk | data are reported only for CROP and wait list. Data for CBT and EMDR groups are reported only in graphs |
| Other bias | Unclear risk | QUOTE: "all the instruments were administered in their written Russian version by the first author after being translated and back translated by professional interpreters. All participants were able to read and understand the questionnaires" |

## Stenmark 2013

| **Bias** | **Authors' judgement** | **Support for judgement** |
| --- | --- | --- |
| Random sequence generation (selection bias) | Low risk | QUOTE: "Participants were randomized to the treatment conditions by drawing ball from a bag with an a-priori 2/3 chance of receiving NET and 1/3 chance of receiving TAU" |
| Allocation concealment (selection bias) | Unclear risk | no information provided |
| Blinding of participants and personnel (performance bias) | High risk | participants not blind and personnel cannot be blind for this type of treatment |
| Blinding of outcome assessment (detection bias) | Low risk | single blind: outcomes assessor. QUOTE: "assessor had no access to information about what therapy the patients' had been assigned to and the therapists were instructed not to reveal the type of treatment their patients were given. The aim was to make the assessor as blind as possible to the patients' treatments." |
| Incomplete outcome data (attrition bias) | Unclear risk | authors state that intention-to-treat analyses were conducted and that results did not differ from treatment completers. However only completers results are reported. Drop-out were balanced in two groups |
| Selective reporting (reporting bias) | High risk | data are reported only in graphs |
| Other bias | Unclear risk | there are differences in the background training of the therapists. Patients were heterogeneous in terms of country of origin. QUOTE: "assessment tools were not validated to the language and culture of each participant". Two of the authors of the paper are authors of the NET manual |

## Weine 2008

| **Bias** | **Authors' judgement** | **Support for judgement** |
| --- | --- | --- |
| Random sequence generation (selection bias) | Unclear risk | QUOTE: "subjects were randomly assigned to one of the two conditions" |
| Allocation concealment (selection bias) | Unclear risk | no information provided |
| Blinding of participants and personnel (performance bias) | High risk | participants not blind and personnel cannot be blind for this type of treatment |
| Blinding of outcome assessment (detection bias) | Unclear risk | no details are provided on how the outcome were assessed |
| Incomplete outcome data (attrition bias) | Unclear risk | no information provided |
| Selective reporting (reporting bias) | High risk | most of the outcomes were not reported as raw data but only as random effects model |
| Other bias | Unclear risk | all participants were Bosnian. QUOTE: "all instruments were translated into Bosnian by the research team. Back translations were used to improve the word selection and to verify that questions were understandable to the refugees." |
