# Supplementary material for: Psychosocial interventions for post-traumatic stress disorder in refugees and asylum seekers resettled in high-income countries: Systematic review and meta-analysis
Source: PLoS One. 2017 Feb 2;12(2):e0171030. doi: 10.1371/journal.pone.0171030 (PMC5289495; doi:10.1371/journal.pone.0171030)
Supplement: S7 Table — (DOCX) [file pone.0171030.s007.docx]

# S7 Table. GRADE summary of findings table

:

|  | Quality assessment | | | | | | | № of patients | Effect | | Confidence in estimate |
| --- | --- | --- | --- | --- | --- | --- | --- | --- | --- | --- | --- |
| Type of intervention | **Outcome** | **Studies** | **Risk of bias** | **Inconsistency** | **Indirectness** | **Imprecision** | **Other considerations** | **Sample size** | **NNT** | **SMD**  (lower scores favours psychosocial interventions) |  |
| NET | PTSD symptoms | 5 RCTs | not serious ^1^ | not serious | not serious | serious ^2^ | asymmetrical funnel plot | 187 | 6.7 | **-0.78**  (-1.18 to -0.38) | ⨁⨁◯◯ LOW |
|  | Depressive symptoms | 3 RCTs | serious ^3^ | serious ^4^ | not serious | serious ^2^ | none | 116 | 4.3 | **-0.86**  (-1.65 to -0.06) | ⨁◯◯◯ VERY LOW |
| CBT | PTSD symptoms | 4 RCTs | serious ^5^ | very serious ^6^ | not serious | serious ^7^ | asymmetrical funnel plot | 182 | - | **-0.97**  (-2.2 to 0.26) | ⨁◯◯◯ VERY LOW |
|  | Depressive symptoms | 3 RCTs | serious ^8^ | very serious ^9^ | not serious | serious ^7^ | none | 152 | - | **-1.54**  (-3.38 to 0.29) | ⨁◯◯◯ VERY LOW |
| EMDR | PTSD symptoms | 0 studies |  |  |  |  |  |  | - | **-** | - |
|  | Depressive symptoms | 0 studies |  |  |  |  |  |  | - | **-** | - |
| TFP | PTSD symptoms | 2 CCTs | very serious ^10^ | very serious ^11^ | not serious | serious ^2^ | none | 146 | 2.1 | **-1.92**  (-3.05 to -0.8) | ⨁◯◯◯ VERY LOW |
|  | Depressive symptoms | 1 CCT | very serious ^10^ | not serious ^12^ | not serious | serious ^2^ | none | 82 | 3.3 | **-1.04**  (-1.59 to -0.5) | ⨁◯◯◯ VERY LOW |
| CROP | PTSD symptoms | 1 RCT | serious ^13^ | not serious ^12^ | not serious | very serious ^14^ | none | 28 | - | **-0.41**  (-1.17 to 0.34) | ⨁◯◯◯ VERY LOW |
|  | Depressive symptoms | 1 RCT | serious ^13^ | not serious ^12^ | not serious | serious ^15^ | none | 28 | 3.7 | -1.1  (-1.67 to -0.54) | ⨁⨁◯◯ LOW |

**NET**: Narrative exposure therapy; **CBT**: Cognitive behavioural therapy; **EMDR**: Eye movement desensitization and reprocessing; **TFP**: Trauma focused psychotherapy; **CROP**: Culture-Sensitive Oriented Peer; **CI:** Confidence interval; **SMD:** Standardised mean difference; NNT: number-needed-to-be-treated. FOOTNOTES: 1: participants and clinicians are not blind to group allocation; however, the outcome assessor was blind in 4 out of 5 studies; 2: overall number of patients included in the analysis is low (less than 200); 3: outcome assessment is not masked in 1 out of 3 studies included in the analysis, and dropouts are not similarly distributed across treatment arms in 1 out of 3 studies; 4: I squared = 70%; 5: participants and clinicians are not blind to group allocation in 3 out of 4 studies, and outcome assessor is not blind in 2 out of 4 studies, and data on dropout are not reported in 2 out of 4 studies; 6: I squared = 91%; 7: overall number of patients included in the analysis is low (less than 200) and CI ranges from appreciable benefit with CBT to no difference; 8: participants and clinicians are not blind to group allocation in 2 out of 3 studies, and outcome assessor is not blind in 1 out of 3 studies, and data on dropout are not reported in 2 out of 3 studies; 9: I squared = 93%; 10: study design is not randomised; 11: I squared = 85%; 12: not applicable: only one study included in the analysis; 13: participants and clinicians are not blind to group allocation, and no information is provided on blinding of outcome assessor, and high number of dropouts; 14: overall number of patients included in the analysis is very low (N=28), and CI ranges from benefit with CROP to no effect; 15: overall number of patients included in the analysis is very low (N=28)
