# Supplementary material for: Psychosocial interventions for post-traumatic stress disorder in refugees and asylum seekers resettled in high-income countries: Systematic review and meta-analysis
Source: PLoS One. 2017 Feb 2;12(2):e0171030. doi: 10.1371/journal.pone.0171030 (PMC5289495; doi:10.1371/journal.pone.0171030)
Supplement: S1 Fig — (DOCX) [file pone.0171030.s008.docx]

# S1 Fig. Publication bias

According to Duval and Tweedie’s trim and fill procedure there are relatively few studies falling toward the right of the mean effect (see funnel plot below), raising a concern that these right-hand studies may actually exist, and are missing from the analysis. Duval and Tweedie’s procedure allows to impute these studies to determine where the missing studies are likely to fall (black dots), add them to the analysis, and then re-compute the combined effect, as reported in the Funnel plot below.
