# Supplementary material for: Psychosocial interventions for post-traumatic stress disorder in refugees and asylum seekers resettled in high-income countries: Systematic review and meta-analysis
Source: PLoS One. 2017 Feb 2;12(2):e0171030. doi: 10.1371/journal.pone.0171030 (PMC5289495; doi:10.1371/journal.pone.0171030)

# S2 Fig. Impact of each study on the pooled effect (sensitivity analysis): consecutively removal of each study as a possible outlier to test what the impact is on the combined effect


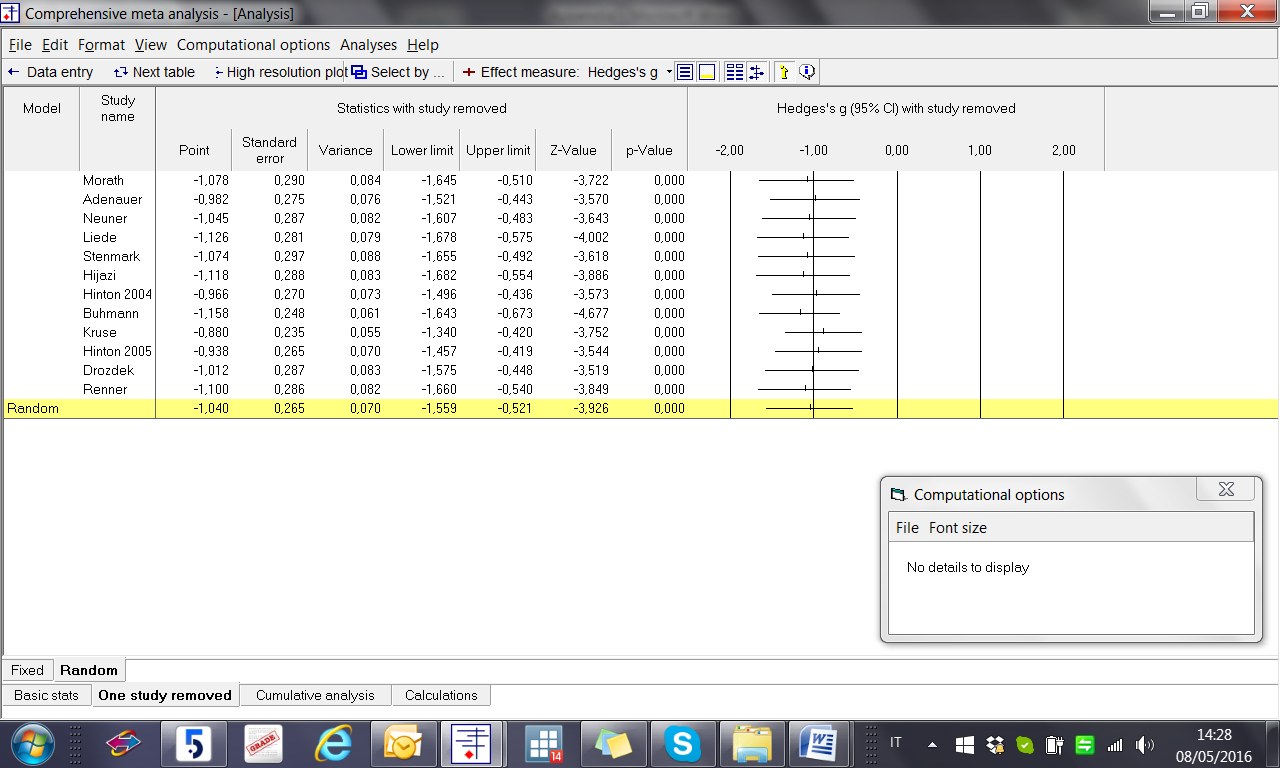

Supplement: S2 Fig — (DOCX) [file pone.0171030.s009.docx]
