# Supplementary material for: Psychosocial interventions for post-traumatic stress disorder in refugees and asylum seekers resettled in high-income countries: Systematic review and meta-analysis
Source: PLoS One. 2017 Feb 2;12(2):e0171030. doi: 10.1371/journal.pone.0171030 (PMC5289495; doi:10.1371/journal.pone.0171030)
Supplement: S3 Fig — (DOCX) [file pone.0171030.s010.docx]

# S3 Fig. Forest plot of secondary outcomes not reported in the main report

Forest plot of comparison: 1 Psychological interventions in PTSD, outcome: PTSD symptoms at follow-up.

Forest plot of comparison: 1 Psychological interventions in PTSD, outcome: PTSD diagnosis at follow-up.

Forest plot of comparison: 1 Psychological interventions in PTSD, outcome: Dropout rate.
