# Supplementary material for: Psychosocial interventions for post-traumatic stress disorder in refugees and asylum seekers resettled in high-income countries: Systematic review and meta-analysis
Source: PLoS One. 2017 Feb 2;12(2):e0171030. doi: 10.1371/journal.pone.0171030 (PMC5289495; doi:10.1371/journal.pone.0171030)

# S4 Fig. Forest plot of subgroup analysis – PTSD symptoms

Forest plot of comparison: 2 PTSD symptoms subgroup analyses, outcome: 2.1 PTSD symptoms by interventions.


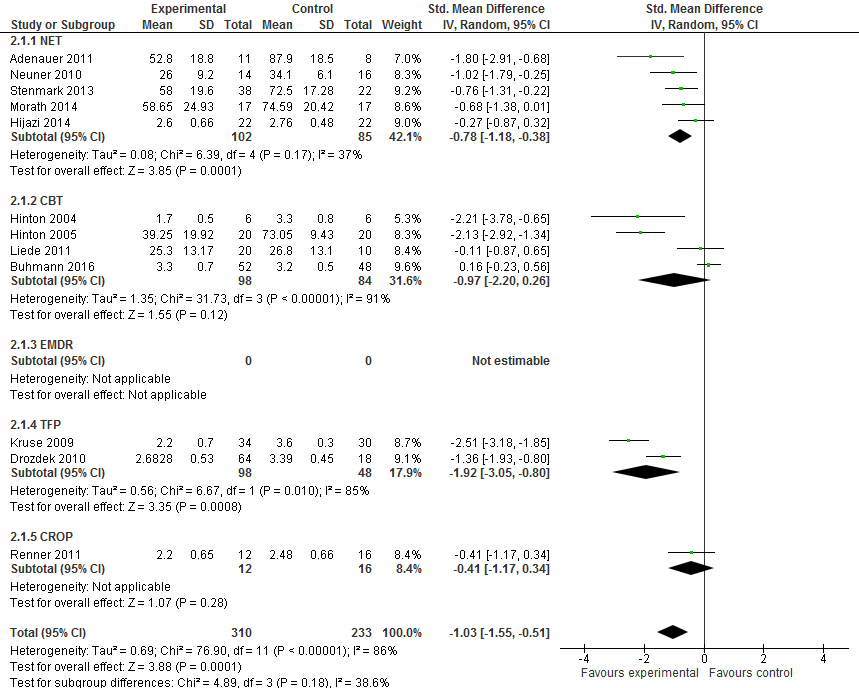


Forest plot of comparison: 2 PTSD symptoms subgroup analyses, outcome: 2.2 PTSD symptoms by study design.


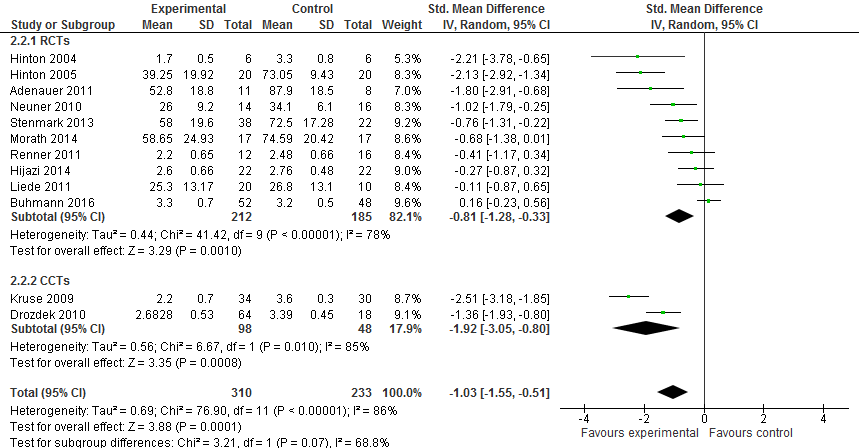


Forest plot of comparison: 2 PTSD symptoms subgroup analyses, outcome: 2.3 PTSD symptoms by study quality.


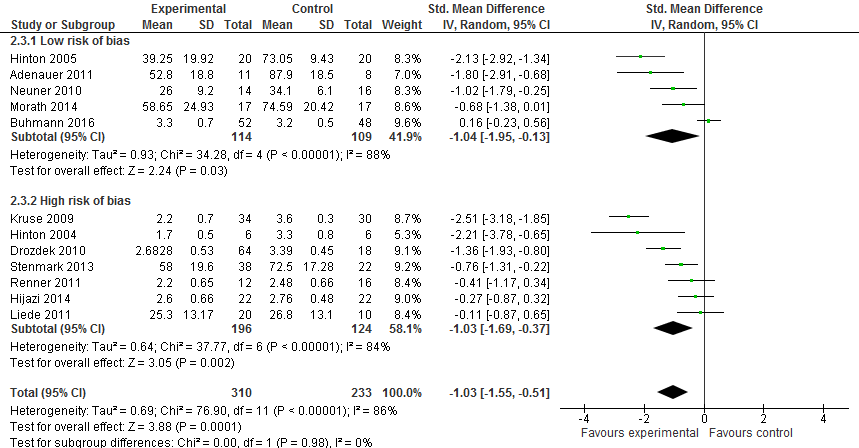


Forest plot of comparison: 2 PTSD symptoms subgroup analyses, outcome: 2.4 PTSD symptoms by rating scale.


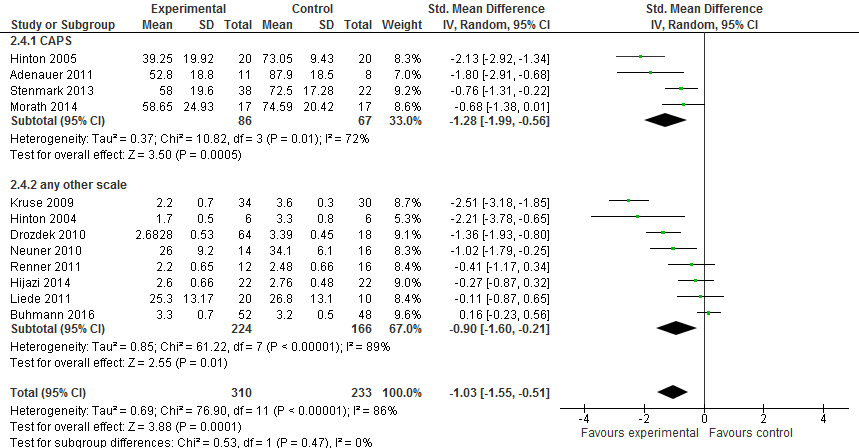


Forest plot of comparison: 2 PTSD symptoms subgroup analyses, outcome: 2.5 PTSD symptoms by number of sessions.


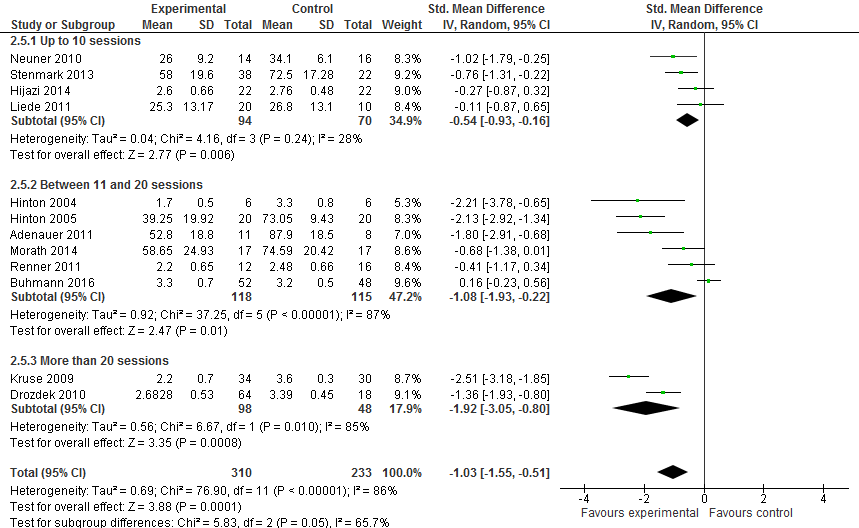


Forest plot of comparison: 2 PTSD symptoms subgroup analyses, outcome: 2.6 PTSD symptoms by length of follow-up.


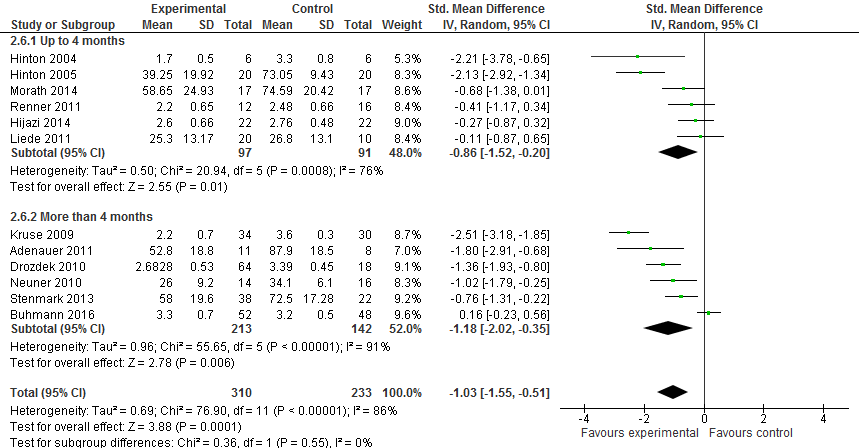


Forest plot of comparison: 2 PTSD symptoms subgroup analyses, outcome: 2.7 PTSD symptoms by country.


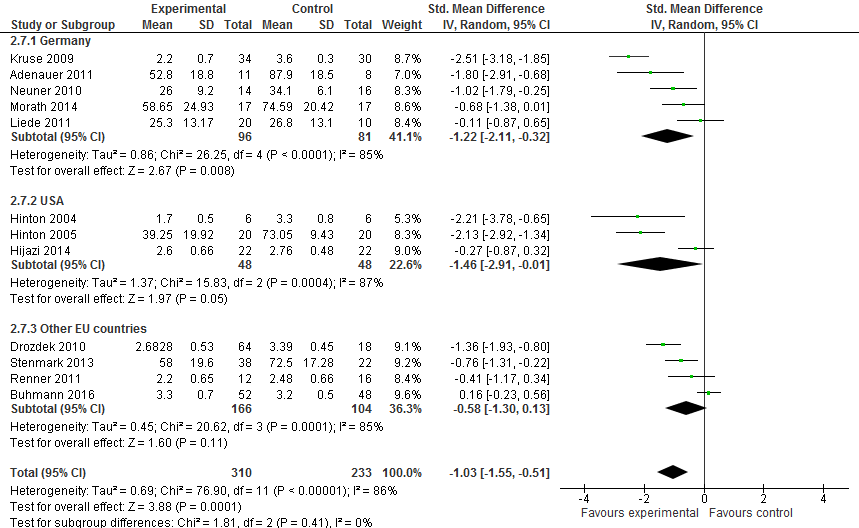


Forest plot of comparison: 2 PTSD symptoms subgroup analyses, outcome: 2.8 PTSD symptoms by ethnicity.


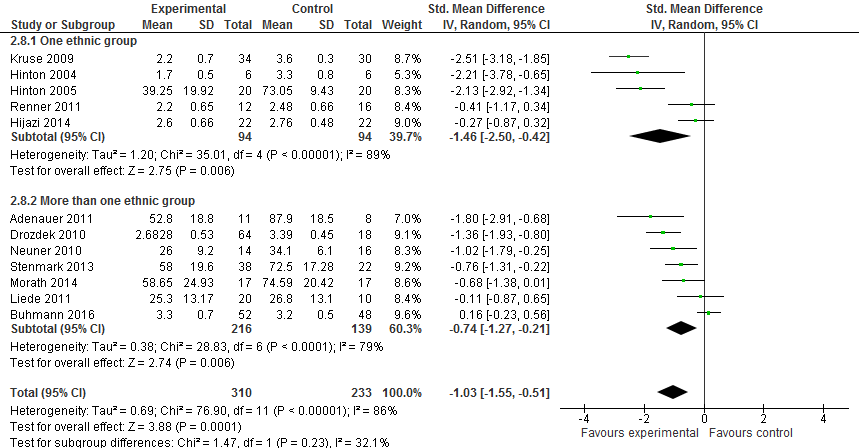

Supplement: S4 Fig — (DOCX) [file pone.0171030.s011.docx]
