# Supplementary material for: Psychosocial interventions for post-traumatic stress disorder in refugees and asylum seekers resettled in high-income countries: Systematic review and meta-analysis
Source: PLoS One. 2017 Feb 2;12(2):e0171030. doi: 10.1371/journal.pone.0171030 (PMC5289495; doi:10.1371/journal.pone.0171030)

# S6 Fig. Unrestricted maximum likelihood random effects meta-regression analysis investigating the association between length of follow-up and effect size (Hedges’s g)

**Regression of followup on Hedges's g**

**Follow-up**

**Hedges's g**

**2**

**3**

**4**

**5**

**6**

**7**

**8**

**9**

**10**

**0,20**

**-0,12**

**-0,44**

**-0,76**

**-1,08**

**-1,40**

**-1,72**

**-2,04**

**-2,36**

**-2,68**


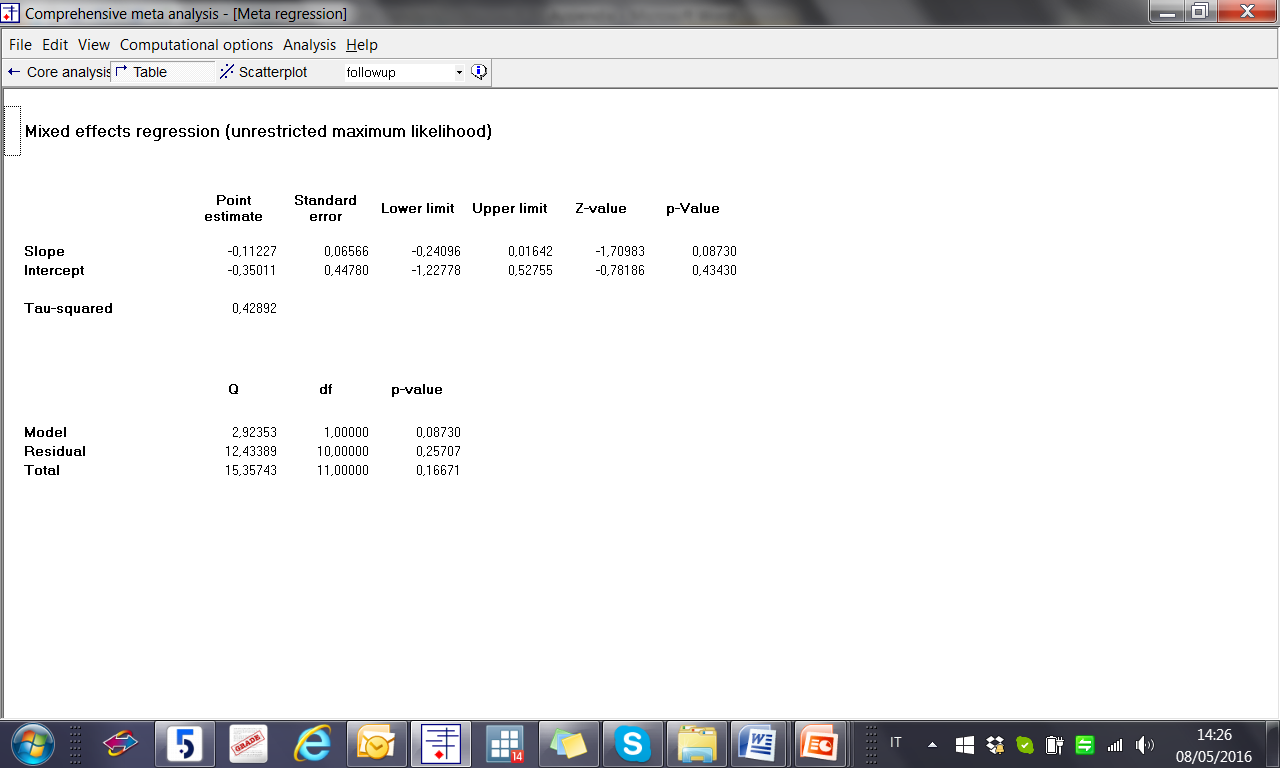

Supplement: S6 Fig — (DOCX) [file pone.0171030.s013.docx]
