# Supplementary material for: Psychosocial interventions for post-traumatic stress disorder in refugees and asylum seekers resettled in high-income countries: Systematic review and meta-analysis
Source: PLoS One. 2017 Feb 2;12(2):e0171030. doi: 10.1371/journal.pone.0171030 (PMC5289495; doi:10.1371/journal.pone.0171030)

# S7 Fig. Forest plot of subgroup analysis – Depressive symptoms

Forest plot of comparison: 3 Depressive symptoms subgroup analyses, outcome: 3.1 Depressive symptoms by interventions.


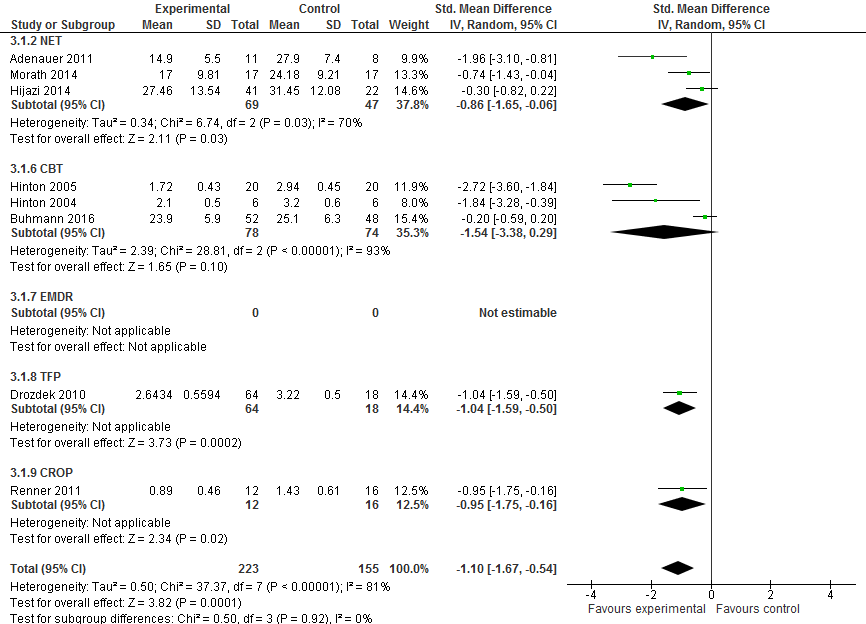


Forest plot of comparison: 3 Depressive symptoms subgroup analyses, outcome: 3.2 Depressive symptoms by study design.


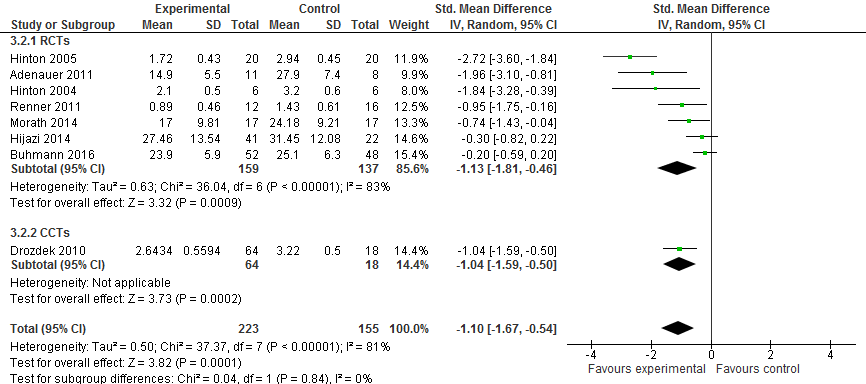


Forest plot of comparison: 3 Depressive symptoms subgroup analyses, outcome: 3.3 Depressive symptoms by study quality.


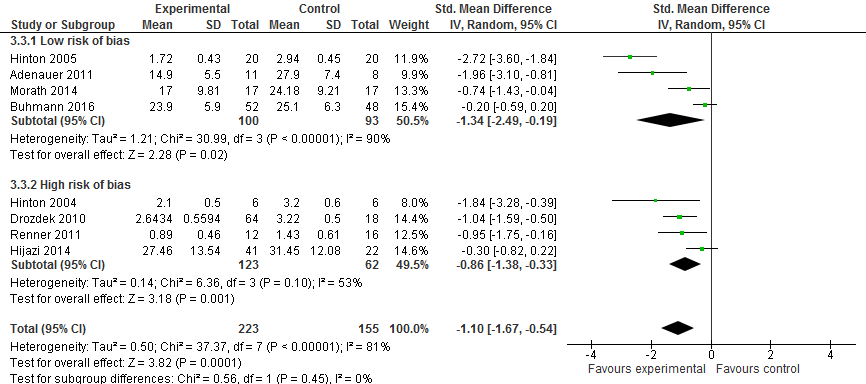


Forest plot of comparison: 3 Depressive symptoms subgroup analyses, outcome: 3.4 Depressive symptoms by rating scale.


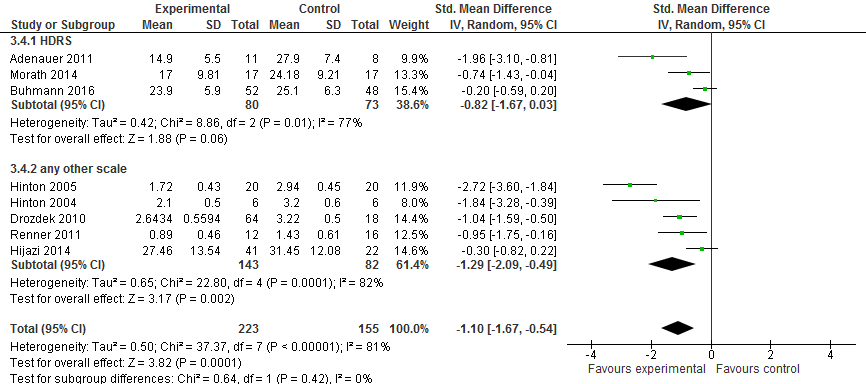


Forest plot of comparison: 3 Depressive symptoms subgroup analyses, outcome: 3.5 Depressive symptoms by number of sessions.


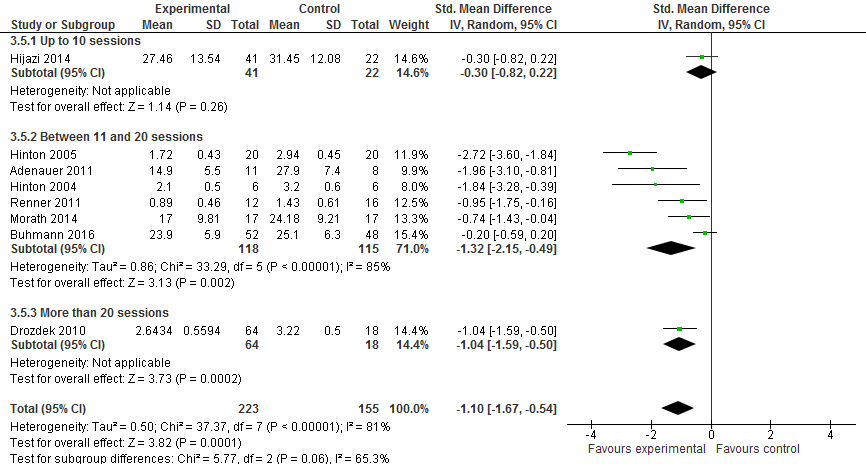


Forest plot of comparison: 3 Depressive symptoms subgroup analyses, outcome: 3.6 Depressive symptoms by length of follow-up.


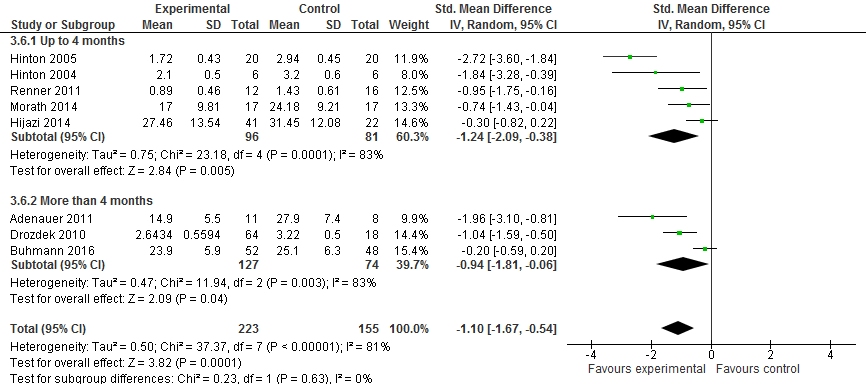


Forest plot of comparison: 3 Depressive symptoms subgroup analyses, outcome: 3.7 Depressive symptoms by country.


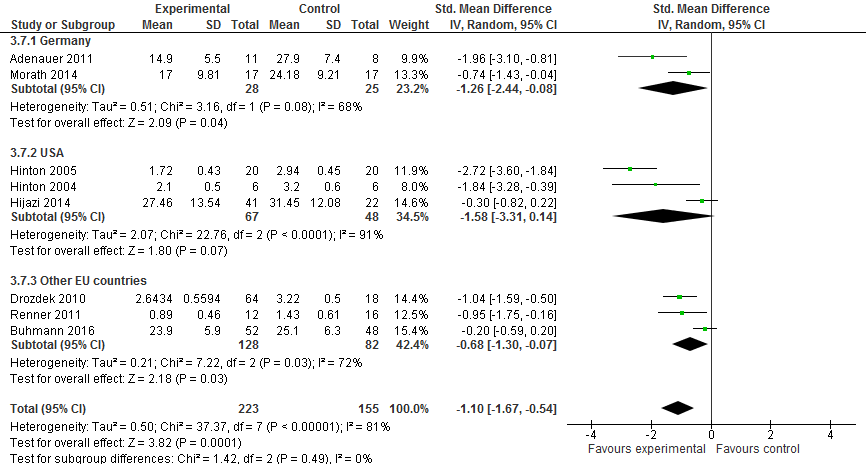


Forest plot of comparison: 3 Depressive symptoms subgroup analyses, outcome: 3.8 Depressive symptoms by ethnicity.


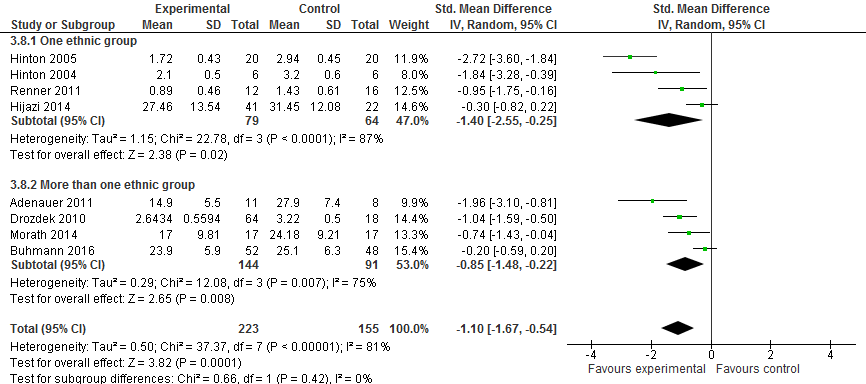

Supplement: S7 Fig — (DOCX) [file pone.0171030.s014.docx]
